# PepH3-modified nanocarriers for delivery of therapeutics across the blood-brain barrier

**DOI:** 10.1186/s12987-025-00641-0

**Published:** 2025-04-01

**Authors:** Anikó Szecskó, Mária Mészáros, Beatriz Simões, Marco Cavaco, Catarina Chaparro, Gergő Porkoláb, Miguel A.R.B. Castanho, Mária A. Deli, Vera Neves, Szilvia Veszelka

**Affiliations:** 1 Biological Barriers Research Group, Institute of Biophysics, HUN-REN Biological Research Centre, Szeged, Hungary; 2 Doctoral School of Biology, University of Szeged, Szeged, Hungary; 3 Instituto de Medicina Molecular, Faculdade de Medicina, Universidade de Lisboa, Lisboa, Portugal; 4 One Health Institute, Faculty of Health Sciences, University of Debrecen, Nagyerdei krt. 98, Debrecen, H-4032 Hungary

**Keywords:** Blood-brain barrier, Brain delivery, Nanoparticle, PepH3, Single domain antibody

## Abstract

**Background:**

Nanocarriers targeting the blood-brain barrier (BBB) are promising drug delivery systems to enhance the penetration of therapeutic molecules into the brain. Immunotherapy, particularly monoclonal antibodies designed to bind amyloid-beta peptides have become a promising strategy for Alzheimer’s disease, but ensuring efficacy and safety is challenging and crucial for these therapies. Our aim was to develop an innovative nanocarriers conjugated with PepH3, a cationic peptide derived from Dengue virus type-2 capsid protein that crosses the BBB and acts as a shuttle peptide for the encapsulated single domain antibody (sdAb) recognizing Aβ oligomers.

**Results:**

PepH3 peptide enhanced the uptake of the nanoparticles (NPs) into brain endothelial cells, and transcytosis of sdAb, as a potential therapeutic molecule, across both rat and human BBB culture models. The cargo uptake was a temperature dependent active process that was reduced by metabolic and endocytosis inhibitors. The cellular uptake of the cationic PepH3-tagged NPs decreased when the negative surface charge of brain endothelial cells became more positive after treatments with a cationic lipid or with neuraminidase by digesting the glycocalyx. The NPs colocalized mostly with endoplasmic reticulum and Golgi apparatus and not with lysosomes, indicating the cargo may avoid cellular degradation.

**Conclusions:**

Our results support that combination of NPs with a potential brain shuttle peptide such as PepH3 peptide can improve the delivery of antibody fragments across the BBB.

**Supplementary Information:**

The online version contains supplementary material available at 10.1186/s12987-025-00641-0.

## Background

Brain diseases, such as Alzheimer’s, Parkinson’s, and various mental disorders, still pose a public health challenge with significant impact on society, affecting healthcare systems, communities, and individuals’ well-being. Drug delivery to the brain is difficult, as most drug candidates developed for neurological diseases have limited access to brain targets due to the blood-brain barrier (BBB) [1]. To increase drug delivery and to avoid degradation in the circulation, a possible approach is to encapsulate therapeutic molecules into nanocarriers [2–4].

Non-ionic surfactant based nanovesicles called niosomes, offer several advantages for therapeutics’ delivery to the brain. Niosomes are biocompatible, biodegradable and less likely to induce adverse effects. These nanoparticles (NPs) can be easily modified to encapsulate both hydrophilic and hydrophobic therapeutic agents, including antibodies, but also to release them in a controlled manner, reducing frequency of administration [5]. Encapsulation of therapeutic antibodies within NPs, not only provides a protective shield against degradation and immune recognition, but also enhances their solubility and, consequently, bioavailability. To mitigate off-target toxicity, niosomes can also be designed to target the brain by surface functionalization with targeting moieties, that specifically interact with brain endothelial cells, as previously showed by our group [6–8]. Peptides that facilitate the passage of drug molecules or nanocarrier systems across the BBB are called BBB peptide shuttles (BBBpS). Drug-conjugated BBBpS or nanocarriers tagged with BBB-specific biomolecules enhance drug delivery across the BBB by utilizing physiological transport pathways, including carrier-mediated, receptor-mediated or adsorptive-mediated transcytosis [9, 10].

The cationic 7-residue PepH3 peptide (AGILKRW), a BBBpS derived from Dengue virus type-2 capsid protein, previously reported by our group, showed an effective penetration across culture BBB models by adsorptive-mediated transcytosis [11, 12]. Furthermore, our in vivo biodistribution data with radiolabeled PepH3 peptide derivatives showed high brain uptake [11]. Compared to the biodistribution of FC5, an antibody fragment that engages receptor-mediated transport, the in vivo biodistribution profile of PepH3 shows low accumulation in liver, lung and kidney [11]. There was a fast clearance from the brain and high levels of excretion, showing that PepH3 is a suitable candidate to be used as a peptide shuttle taking cargo into the central nervous system (CNS) [11]. PepH3 peptide conjugated to a triple-negative breast cancer (TNBC)-specific anticancer peptide motif (vCPP2319) crossed cell culture models of the BBB, penetrated into the brain in vivo, and showed serum stability with a half-life above 120 min [13]. In the same way, PepH3 peptide linked to the 3-dimensional self-assembled palladium based metallacages successfully translocated across the BBB both in vitro and in vivo in mice [14].

Immunotherapy, particularly monoclonal antibodies (mAbs), have become a promising avenue for brain diseases, including Alzheimer’s disease (AD). They are designed to bind to specific targets in the brain, such as pathogenic proteins or molecules, minimizing off-target effects, promoting clearance mechanisms, or modulating neuroinflammation [15, 16]. In AD, mAbs, such as aducanumab, lecanemab, both FDA approved, targeting soluble forms of amyloid-beta (Aβ) aggregates, have entered clinical trials. However, most of these anti-Aβ mAbs therapies did not meet primary endpoints, failing to show clinically relevant effects in patients [17]. The limited efficacy of these approach can be associated with immunogenicity, low long-term antibody stability associated with degradation and aggregation over time, which can reduce effectiveness and safety and low BBB penetration due to highly selective transport mechanisms [18, 19]. Overcoming these limitations is crucial for ensuring the efficacy and safety of antibody-based therapies. We selected as a model biomolecule cargo a single domain antibody (sdAb) recognizing Aβ oligomers [18, 20].

The major aim of the study was to prove that PepH3 that successfully shuttled bioconjugates across the BBB both in vitro and in vivo, is also able to increase the internalization and BBB crossing of NPs with sdAb as a biomolecule cargo. Specifically, we designed, characterized and investigated an innovative PepH3-tagged vesicular nanocarrier to cross the BBB and act as a shuttle for the encapsulated biomolecule.

## Materials and methods

### Animals

For primary cell isolations, brain tissues were obtained from 3-week-old and newborn outbred Wistar rats (Harlan Laboratories, Envigo, Indianapolis, IN, USA) of both sexes. The animals were fed on standard rodent chow and water *ad libitum* and were kept under a 12 h light/dark cycle in the conventional animal house of the Biological Research Centre, Szeged. Organ harvest from animals was performed following the regulations of the 1998 XXVIII Hungarian law and the EU Directive 2010/63/EU about animal protection and welfare.

### Materials

All reagents were purchased from Merck Life Science Kft., Budapest, Hungary, unless otherwise indicated. PepH3 peptide labeled by Quasar 570 dye (Q-AGILKRW-NH2; 1.3 kDa) was a kind gift from David Andreu Group (PRBB, Barcelona). DSPE-PEG(2000)-PepH3 peptides was purchased from JPT Peptide Technologies GmbH, Germany. His-tagged single domain antibody (sdAb) anti-Aβ 1–42 was purchased from VectorB2B, Portugal [20].

### Preparation of PepH3 tagged nanovesicles

Vesicular NPs were made from non-ionic surfactants and cholesterol. For the preparation of non-tagged nanovesicles (N), Span 60 (sorbitane-monostearate), Solulan C24 (cholesterylpoly-24-oyxyethylene-ether, Chemron Co., USA) and cholesterol were dissolved in hot 1:2 mixtures of chloroform and ethanol in a round-bottom flask [6, 7, 21]. To prepare DSPE-PEG-PepH3 tagged NPs (N-PepH3) polyethyleneglycol conjugated PepH3 peptide (4.5 w/w% of total lipids) was added to the mixture before the dissolving step. Removal of organic solvents by vacuum pump yielded a thin lipid film layer. The dry lipid film was hydrated with phosphate buffer (PBS; KCl 2.7 mM, KH_2_PO_4_ 1.5 mM, NaCl 136 mM, Na_2_HPO_4_ × 2 H_2_O 6.5 mM, pH 7.4) containing 0.1 mg/mL Texas red-labeled bovine serum albumin (TR-BSA, 67.12 kDa, Thermo Fischer Scientific, USA) or 0.186 mg/mL single domain antibody (sdAb, 14.03 kDa) as cargo. The mixture was heated at 45 ^o^C in a water bath and sonicated for 1 h. The suspension was filtered through a syringe filter with 0.45 μm and 0.2 μm pore size (Sarstedt, Germany) to yield vesicles. The non-entrapped cargo was removed by ultracentrifugation (123,249 *g*, 3 h, 4 ^o^C), which resulted in a supernatant and a wet pellet on the bottom of the tube. The wet pellet contains the hydrated lipid bilayers that form the niosomal structure. After we removed precisely and carefully the aqueous supernatant phase by using micropipette and thin strips of sterile filter paper, the mass of the remaining wet pellet was measured. To prepare a 100 mg/mL concentration of NPs we added the required amount of gentamycin-containing, phenol red-free DMEM/HAM’s F12 culture medium (Gibco, USA) to the pellet and resuspended it. The nanovesicles were either freshly used or stored at 4 °C until further experiments.

### Encapsulation efficiency of NPs (EE %)

The amount of encapsulated cargo in the synthesized NPs was determined by indirect method. Briefly, the non-entrapped cargo was detected in the supernatant from the last ultracentrifugation step, by spectrofluorometry (Fluorog 3, Horiba Jobin Yvon, France) at 589/609 nm, for TR-BSA. Anti-His6 antibody (Roche, Germany) was used for detection of sdAb by Western Blot. The encapsulation efficiency percentage (EE %) was calculated by the following equation:

$$\:\text{E}\text{E}\text{\%}=\frac{\begin{array}{c}Initial\:total\:amount\:of\:cargo-\\\:Amount\:of\:cargo\:in\:the\:supernatant\end{array}}{\begin{array}{c}Initial\:total\:\\\:amount\:of\:cargo\end{array}}\times\:100$$

### Size and surface charge measurements

To characterize the physico-chemical properties, NPs were diluted in PBS or distilled water to a final concentration of 5 mg/mL. The hydrodynamic size, polydispersity index (PDI) and surface charge (zeta potential) of NPs were measured by dynamic light scattering (DLS) (Malvern Zetasizer Ultra, UK). Stability and protein corona formation were also measured with Malvern Zetasizer on NPs with sdAb cargo.

### Protein Corona and stability measurements

Protein corona formation was evaluated in a time dependent manner by incubation of the NPs in human plasma (dilution ratio: 1:1) for 10 min, 30 min, 1 h, 3 and 24 h, at 37 ºC. Prior to DLS and zeta potential measurements, niosomes were washed twice with PBS by centrifugation at 14 000 rpm for 10 min, and resuspended in PBS. Mean values were calculated from the average of triplicates.

For stability measurements, NPs were diluted in DMEM/HAM’s F12 (Gibco, USA) at pH 7.4 or PBS at pH 7.4 or pH 5, and incubated at 37 ºC. To assess stability, the size and PDI were measured by DLS at 0 min and after 24 h.

### Cell cultures

Isolation of primary rat brain endothelial cells (RBEC), pericytes (RPC) and astrocytes (RAC) were performed according to the method described in our previous studies [8, 22]. After isolation, brain endothelial cells were seeded onto culture dishes (Corning Costar, USA) coated with collagen type IV (100 µg/mL) and fibronectin (25 µg/mL) and were cultured in DMEM/HAM’s F12 supplemented with 15% plasma-derived bovine serum (PDS, First Link, UK), 10 mM HEPES, 100 µg/mL heparin, 5 µg/mL insulin, 5 µg/mL transferrin, 5 ng/mL sodium selenite (ITS, Pan-Biotech, Germany), 1 ng/mL basic fibroblast growth factor (bFGF, Roche, Switzerland) and 50 µg/mL gentamicin. During the first 3 days of culture, the medium of endothelial cells also contained 3 µg/mL puromycin to eliminate P-glycoprotein negative, contaminating cell types [23]. After the first 3 days of culture, the amount of PDS was decreased from 15 to 10% in the culture medium of RBECs.

After isolation, RPC were seeded onto culture dishes (VWR International, USA) coated with collagen type IV (100 µg/mL), whereas RAC were plated onto uncoated 75 cm^2^ flasks (TPP, Switzerland). Both RPC and RAC were cultured in DMEM medium (low glucose, Gibco, USA) supplemented with 10% fetal bovine serum (FBS, Pan-Biotech, Germany) and 50 µg/mL gentamicin.

Human cerebral microvascular endothelial cells HBEC-5i (ATCC^®^ CRL-3245™) were cultured as a monolayer on gelatin-coated T-Flasks (Corning Costar, USA) using DMEM/HAM’s F12 supplemented with 10% FBS (Gibco, USA), 1% penicillin/streptavidin (Gibco, USA), and 40 µg/mL endothelial cell growth supplement (ECGS, Sigma-Aldrich, Spain), according to the manufacturer’s instructions. Cells were grown in a humidified atmosphere of 5% CO_2_ at 37 °C (MCO-18AIC (UV), Sanyo, Japan), with the medium changed every other day.

### Measurement of cellular toxicity

Kinetics of the effect of PepH3 peptide, PepH3-tagged and non-tagged NPs on RBEC cells was monitored by impedance measurement at 10 kHz (RTCA-SP instrument; ACEA Biosciences, USA). Impedance measurement is a label-free, real time, non- invasive method, and correlates linearly with adherence, growth, number, and viability of cells [21]. For background measurements 50 µL cell culture medium was added to the wells, then cells were seeded at a density of 6 × 10^3^ cells/well to collagen type IV (100 µg/mL) and fibronectin (25 µg/mL) coated 96-well plates with integrated gold electrodes (E-plate 96, ACEA Biosciences, USA). Cells were cultured for 4 days and monitored every 5 min until the end of experiments. At the beginning of plateau phase of growth, cells were treated with PepH3 (1.875-120 µM) and PepH3-tagged and non-tagged NPs (0.1-3.0 mg/mL) for 24 h. Cell index was defined as R_n_-R_b_ at each time point of measurement, where R_n_ is the cell-electrode impedance of the well when it contains cells and R_b_ is the background impedance of the well with the medium alone.

The toxicity of NPs on HBEC-5i was determined using the CellTiter-Blue^®^ cell viability assay (Promega, Spain), following a protocol previously optimized [24]. Briefly, HBEC-5i was carefully harvested with trypsin-EDTA and seeded 2 × 10^4^ cells/well into 96-well clear flat bottomed polystyrene plates (Corning Costar, USA). In the case of HBEC-5i, a pre-incubation with gelatin for 1 h is required. After 24 h, the medium was removed, cells were washed twice with PBS, and cells were treated with the NPs (0.2–100 µg/mL) for 24 h. Then, cells were washed twice with PBS and 20 µL of CellTiter-Blue^®^ reagent (diluted in 100 µL medium) was added to each well and incubated for 3 h in culturing conditions. The fluorescence intensity was measured using VarioskanTM LUX multimode microplate reader (ThermoFisher, Spain). Experiments were performed in triplicates on different days using independently grown cell cultures.

### Cellular uptake studies

To visualize the cellular uptake of PepH3 peptide alone, the cells were cultured in Matrigel coated glass bottom culture dishes (diameter: 3.5 cm, Greiner Bio-One, Germany). Confluent monolayers were incubated with the peptide solution (100 nM) diluted in the culture medium of RBEC at 37 °C for 30 min, 5–24 h. For the staining of cell nuclei, Hoechst 33342 dye (1 µg/mL, 10 min) was used. After incubation the culture medium was removed and cells were washed with Ringer-HEPES buffer (118 mM NaCl, 4.8 mM KCl, 2.5 mM CaCl_2_, 1.2 mM MgSO_4_, 5.5 mM D-glucose, 20 mM HEPES, pH 7.4) supplemented with 1% PDS. For visualization of PepH3, living cells was imaged using Leica TCS SP5 confocal laser scanning microscope (Leica Microsystems, Germany).

To investigate the cellular uptake of the NPs, RBECs were cultured in 24-well plates (Corning Costar, USA; 3 × 10^4^ cell/well) coated with collagen type IV (100 µg/mL) and fibronectin (25 µg/mL). The confluent monolayer of RBECs were incubated with N(TR-BSA) or N(TR-BSA)-PepH3 (1 mg/mL) diluted in endothelial cells culture medium for 15 min, 1 and 24 h on a horizontal shaker (150 rpm). After incubations, cells were washed three times with ice cold PBS supplemented with 1% of bovine serum albumin (BSA), once with acid stripping buffer (glycine 50 mM, NaCl 100 mM, pH 3) to remove cell surface-associated NPs and once with PBS. Finally, cells were lysed in distilled water containing 10 mg/mL Triton X-100 detergent. The amount of TR-BSA cargo was quantified with a spectrofluorometer (Horiba Jobin Yvon Fluorolog 3, USA).

To study the uptake mechanisms of tagged nanovesicles with TR-BSA cargo in RBECs, several conditions were tested. Briefly, to observe energy-dependent pathways, RBECs were treated with N-PepH3 (1 mg/mL) at 4 ºC or co-incubated with ATP synthesis blocking sodium azide (1 mg/mL, 24 h). To study the endocytosis, RBECs were pre-treated with two endocytic inhibitors, the lipid raft/caveolin-dependent endocytosis inhibitor filipin (5 µg/mL, 15 min) or the actin polymerization blocking cytochalasin D (0.125 µg/mL, 1 h), then incubated with N-PepH3 (1 mg/mL) at 37 ºC for 24 h. All conditions were compared with the control of RBECs treated with N-PepH3 (1 mg/mL) at 37 ºC for 24 h.

To elucidate the role of glycocalyx of the endothelial cells in the uptake process of the NPs, RBEC cells were pretreated with cationic lipid TMA-DPH (1-(4-trimethylammoniumphenyl)-6-phenyl-1,3,5-hexatriene, 54 µM; Molecular Probes, Life Technologies) for 30 min or digested the sialic acid of glycocalyx with neuraminidase (1 U/mL, 1 h) at 37 ºC.

To track the location of NPs with TR-BSA cargo within the endoplasmic reticulum (ER), Golgi apparatus or lysosomes, RBEC cells were seeded in glass bottom petri dishes (diameter: 3.5 cm, Greiner Bio-One, Germany). The cells were incubated with N or N-PepH3 (1 mg/mL) diluted in culture medium for 24 h. To observe colocalization, cells were co-incubated with selective dyes for ER, Gogi or lysosomes according to the manufacturer’s instructions (ER Staining Kit-Green Fluorescence-Cytopainter Abcam, Cambrige, MA, USA, ab139481, 15 min, 1:1000; Golgi Staining Kit-Green Fluorescence-Cytopainter, Abcam, Cambrige, MA, USA, ab139483, 15 min, 1:100; Lysosomal Staining Kit-Green Fluorescence-Cytopainter, Abcam, Cambrige, MA, USA, ab176826, 30 min, 1:500), and Hoechst 33342 dye (15 min, 1 µg/mL, Thermo Fisher, USA) at 37 °C in a CO_2_ incubator. After incubations, the living cells were washed two times with phenol red-free medium supplemented with 1% PDS, and were imaged using Leica TCS SP5 confocal laser scanning microscope (Leica Microsystems, Germany). These results were calculated by object-recognition based colocalization analysis with pixel-intensity correlation (object-corrected Pearson coefficient) according to the method of Moser et al. [25].

### Permeability study on primary rat co-cultured BBB model (RBEC)

For permeability studies, our well characterized rat triple co-culture BBB model was used, in which RBEC, RPC and RAC are cultured together in a Transwell system [22, 26]. RAC were passaged (8.5 × 10^4^ cells/cm^2^) to collagen type IV (100 µg/mL) coated 24-well plates (Corning Costar, USA) for PepH3 experiments and 12-well plates (Corning Costar, USA) for the NP treatments. To prepare the co-culture model, RPC were passaged (1.5 × 10^4^ cells/cm^2^) to the bottom side of cell culture inserts (Transwell, polycarbonate membrane, 3 µm pore size, Corning Costar, USA) coated with collagen type IV (100 µg/mL). RBEC (7.5 × 10^4^ cells/cm^2^) were seeded to the upper side of the culture insert membrane coated with Matrigel (growth factor reduced, Corning Costar, USA) or Geltrex (517 µg/mL, Thermo Fisher, USA). Then the inserts containing RBEC and RPC on the two sides of the membrane were placed to 12-well or 24-well plates containing RAC at the bottom. Both the upper and lower compartments received endothelial cell culture medium. The 3 cell types were cultured together for 4 days before permeability measurements in culture media supplemented with 550 nM hydrocortisone. Before the permeability experiment, the upper compartment of the model was also supplemented with 250 µM 8-(4-chlorophenylthio) adenosine 3’,5’-cyclic monophosphate (cPT-cAMP) and with 17.5 µM Ro-20-1724, a selective inhibitor of cAMP-specific phosphodiesterase for 24 h to elevate the tightness of the barrier [23, 27].

To verify the integrity of the BBB model, the tightness of the intercellular junctions was measured by an EVOM Voltohmeter (World Precision Instruments, USA) combined with STX2 electrodes, and expressed relative to the surface area of the monolayers (Ω × cm^2^), that reflecting the transendothelial electrical resistance (TEER) of the cells. Resistance of cell-free inserts was subtracted from the measured values. Before the permeability experiments of PepH3 peptide across the BBB model the average TEER value was 292.2 ± 23 Ω × cm^2^ (n = 10), and in the case of the permeability experiments of NPs the average TEER values were 244.9 ± 38 Ω × cm^2^ (n = 26), indicating good barrier properties for both BBB penetration assays.

The donor compartment of BBB model was incubated with either 10 nM PepH3 peptide for 30 min, or 1 mg/mL NPs with sdAb cargo for 24 h, diluted in phenol red-free DMEM/HAM’s F12 medium supplemented with 5% PDS. To assess the integrity of the model, the paracellular marker sodium fluorescein (SF; 376 Da, 10 µg/mL) and the transcellular marker Evans blue albumin (EBA, 67 kDa, 10 mg/mL BSA + 167.5 µg/mL Evans blue) were also tested for permeability. After incubations, samples were collected from the compartments and the fluorescent signal of PepH3 peptide (excitation: 552 nm; emission: 567 nm) was quantified with a spectrofluorometer (Horiba Jobin Yvon Fluorolog 3, USA). The fluorescent signal of the marker molecules was quantified at 485 nm excitation and 520 nm emission wavelengths for SF and 584 nm excitation and 680 nm emission wavelengths for EBA by spectrofluorometer.

For the determination of permeability, the apparent permeability coefficients (P_app_) were calculated with the following equation:

$$\:{\text{P}}_{\text{a}\text{p}\text{p}}\:(\text{c}\text{m}/\text{s})\:=\:\frac{{\left[\text{C}\right]}_{\text{A}}\:\times\:{\text{V}}_{\text{A}}}{\:\text{A}\:\times\:{\left[\text{C}\right]}_{\text{D}}\:\times\:\:\varDelta\:\text{t}}$$

Briefly, P_app_ (cm/s) of PepH3 peptide and the BBB markers, SF and EBA were calculated from the concentration difference of the molecules in the acceptor compartment (Δ[C]_A_) after 30 min. In the case of sdAb loaded NPs, the penetration of markers was measured after 24 h. [C]_D_ is the concentration in the donor compartment at 0 h, V_A_ is the volume of the acceptor compartment in 24 or 12 -wellsplate (900 µL or 1500 µL, respectively), and A is the surface area available for permeability (0.33 cm^2^ in 24 -wellplate and 1.12 cm^2^ in 12 -well plate).

After measuring the BBB permeability of NPs with sdAb cargo, the antibody in the samples was detected by Western blot. To calculate the translocation of sdAb across the BBB model, the intensity of the bands from the acceptor compartment samples was divided by the intensity of the bands from the donor compartments at 0 h and the values were expressed as a percentage of the non-tagged group (N).

### Permeability study on human monocultured cell line-based BBB model (HBEC-5i)

The translocation capacity of the sdAb cargo of NPs was evaluated using an in vitro HBEC-5i cell model, as previously described [24, 28]. Briefly, HBEC-5i cells were carefully harvested with trypsin-EDTA and seeded 8 × 10^3^ cells/well into pre-coated tissue culture inserts (transparent polyester (PET) membrane with 1.0 μm pores) for 24-well plated (Corning Costar, USA). During 8 days, the medium was changed every other day. After medium removal, cells were washed twice with 1X PBS and once with DMEM/HAM’s F12 medium without phenol red. Then, previously diluted NPs (0.1 mg/mL) were added to the apical side of the model and incubated for 24 h. Then, samples were collected from the apical (200 µL/insert) and basolateral (500 µL/insert) side and sdAb was identified by Western blot. Experiments were performed on different days using independently grown cell cultures. The permeability of encapsulated sdAb cargo across the HBEC-5i cells was calculated as described in the case of RBEC model.

After the translocation assay, we evaluated the integrity of the in vitro BBB model, as previously described by our group [24]. Herein, cells were washed twice with PBS and one time with DMEM/HAM’s F12 medium without phenol red. Then, previously diluted fluorescein-dextran 4 kDa (FD4) was added to the apical side and incubated for 2 h. FD4 was diluted in DMEM/HAM’s F12 without phenol red to an absorbance below 0.1. Finally, samples from the basolateral side were collected and fluorescence intensity measured using a VarioskanTM LUX multimode microplate reader. The percentage of FD4 recovered was determined using the following equation:

$$\:\text{T}\text{r}\text{a}\text{n}\text{s}\text{l}\text{o}\text{c}\text{a}\text{t}\text{i}\text{o}\text{n}\:\text{o}\text{f}\:\text{F}\text{D}4\:\left(\text{\%}\right)=\frac{{\text{F}\text{l}\text{u}\text{o}}_{\text{s}\text{a}\text{m}\text{p}\text{l}\text{e}}-{\text{F}\text{l}\text{u}\text{o}}_{\text{c}\text{e}\text{l}\text{l}\text{s}}}{{\text{F}\text{L}\text{u}\text{o}}_{\text{F}\text{D}4}-{\text{F}\text{l}\text{u}\text{o}}_{\text{m}\text{e}\text{d}\text{i}\text{u}\text{m}}}\times\:100$$

### Western blot

Samples from encapsulation efficiency and BBB permeability assays were evaluated by Western blot, using a protocol previously described [29]. Briefly, samples were loaded onto 12% acrylamide Bis-Tris gels and run in Tris/Glycine/SDS running buffer (25 mM Tris, 192 mM Glycine, 0.1% SDS, pH 8.0). The resolved NPs were transferred from the gel onto a nitrocellulose membrane in Tris/Glycine transfer buffer (25 mM Tris, 192 mM Glycine, 20% (v/v) methanol, pH 8.5) at 250 mA for 90 min. Membranes were blocked with 5% low fat milk in 1X PBST (0.1% Twenn 20 in PBS), then blotted with HRP-preadsorbed anti-His (1:500, in 1% low fat milk in 1X PBST) (Roche, USA). The blots were revealed using ECL Prime Western Blotting Detection Reagent (Promega, Spain), according to the manufacturer’s instructions. Then, imaged using the Amersham Imager 680 (GE Healthcare, USA).

### Statistics

Data are presented as means ± SD or SEM. Values were compared using ANOVA following Bonferroni post-tests or unpaired t-test (GraphPadPrism 5.0; GraphPad Software, USA). Changes were considered statistically significant at *P* < 0.05. The number of parallel samples was 4–25.

## Results

### Physico-chemical properties of nanocarriers

**Fig. 1:**
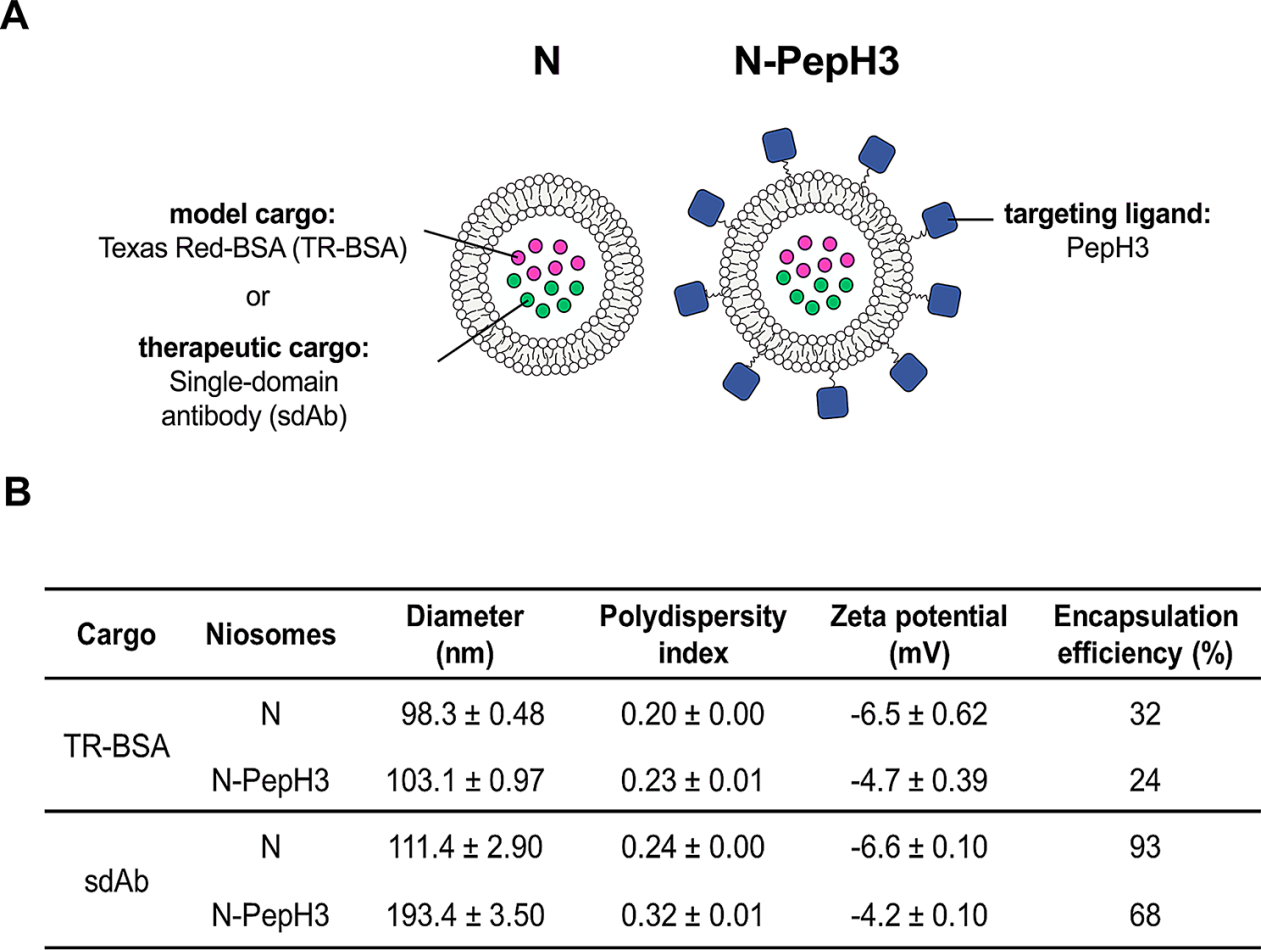
Characterization of the NPs. **A** Schematic drawing of non-tagged and PepH3-tagged NPs with albumin model cargo or single-domain antibody cargo. **B** Main physico-chemical properties of non-tagged and PepH3-tagged niosomes with Texas Red albumin or single-domain antibody cargo. Values presented are means ± SD. N: non-tagged NP; N-PepH3: PepH3-tagged NP; sdAb: single-domain antibody; TR-BSA: Texas Red-labelled bovine serum albumin

Nanocarriers were either non-tagged or PepH3-tagged, and contained fluorescent TR-BSA as a model cargo or single domain antibody (sdAb), as a potential therapeutic cargo (Fig. 1A). The mean diameter of NPs with TR-BSA cargo was very similar, 98 and 103 nm for N and N-PepH3 group, respectively (Fig. 1B). In the case of NPs with sdAb cargo, the diameter of the non-tagged nanocarriers was 111 nm, while the size of the PeH3-tagged NPs increased to 193 nm. In all groups a relatively narrow size distribution was measured as indicated by the polydispersity index (PDI) values below 0.32 (Fig. 1B). Based on the zeta potential measurements, all NPs had slightly negative surface charge. The presence of the positively charged peptide PepH3 made the surface charge of the NPs less negative. The cationic PepH3, as a targeting ligand on the surface of the nanocarriers, increases the zeta potential by ~ 2 mV in a statistically significant way (Supplementary Fig. S1). The encapsulation efficiency of the bigger cargo, the 67 kDa TR-BSA was 32% in the N and 24% in the N-PepH3 groups. In the case of sdAb loaded NPs, the encapsulation efficiency values were higher, 93% and 68% for N and N-PepH3 groups, respectively (Fig. 1B).

### Structural stability of nanovesicles

**Fig. 2:**
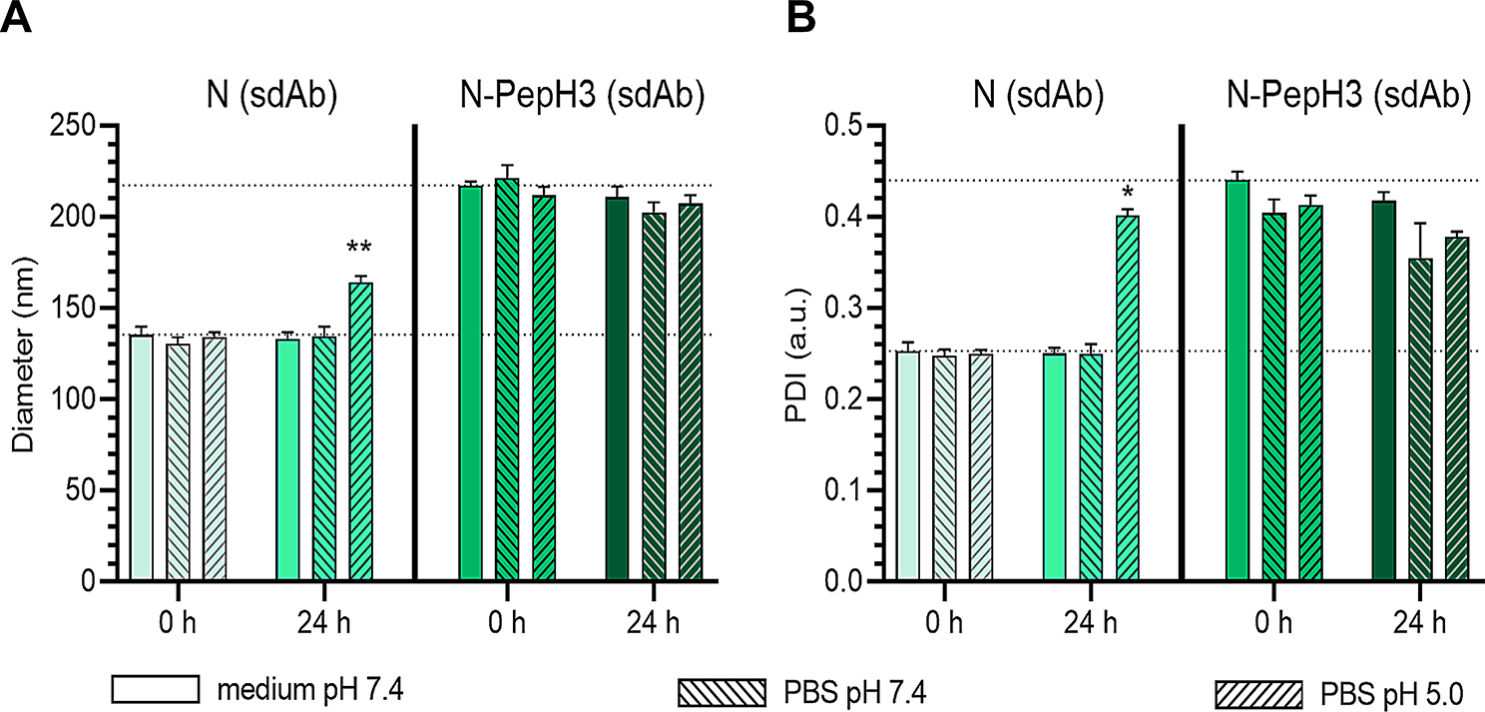
Stability of non-tagged and PepH3-tagged niosomes with single-domain antibody cargo. **A** The mean diameter of the NPs under different conditions. Values presented are means ± SD. Statistical analysis: one-way ANOVA followed by Bonferroni post-test; **p < 0.01 compared to groups in culture medium at pH 7.4 at 0 h; n = 3. **B** Measurement of the polydispersity index of NPs. Values presented are means ± SD. Statistical analysis: one-way ANOVA followed by Bonferroni post-test; *p < 0.05 compared to groups in culture medium at pH 7.4 at 0 h; n = 3. N: non-tagged NP; N-PepH3: PepH3-tagged NP; sdAb: single-domain antibody

To study the stability of NPs under different pH and buffer conditions, the size of the carriers was measured in cell culture medium or PBS at pH 7.4, or at more acidic pH 5 conditions by dynamic light scattering. The tagging ligand on the surface of NPs has increased both the size (Fig. 2A) and PDI (Fig. 2B) values of the N-PepH3 group after 24 h compared to the non-tagged NPs. The characteristic values of the NPs (size and PDI) did not change within the groups under the different conditions, reflecting the good stability of the NPs. The only exception was the N niosome group in PBS at pH 5 after 24 h incubation, where the diameter of NPs and the PDI values were higher compared to other NP groups, indicating lower stability of the non-tagged NPs in acidic buffer after 24 h.

### Protein Corona of the nanoparticles

**Fig. 3:**
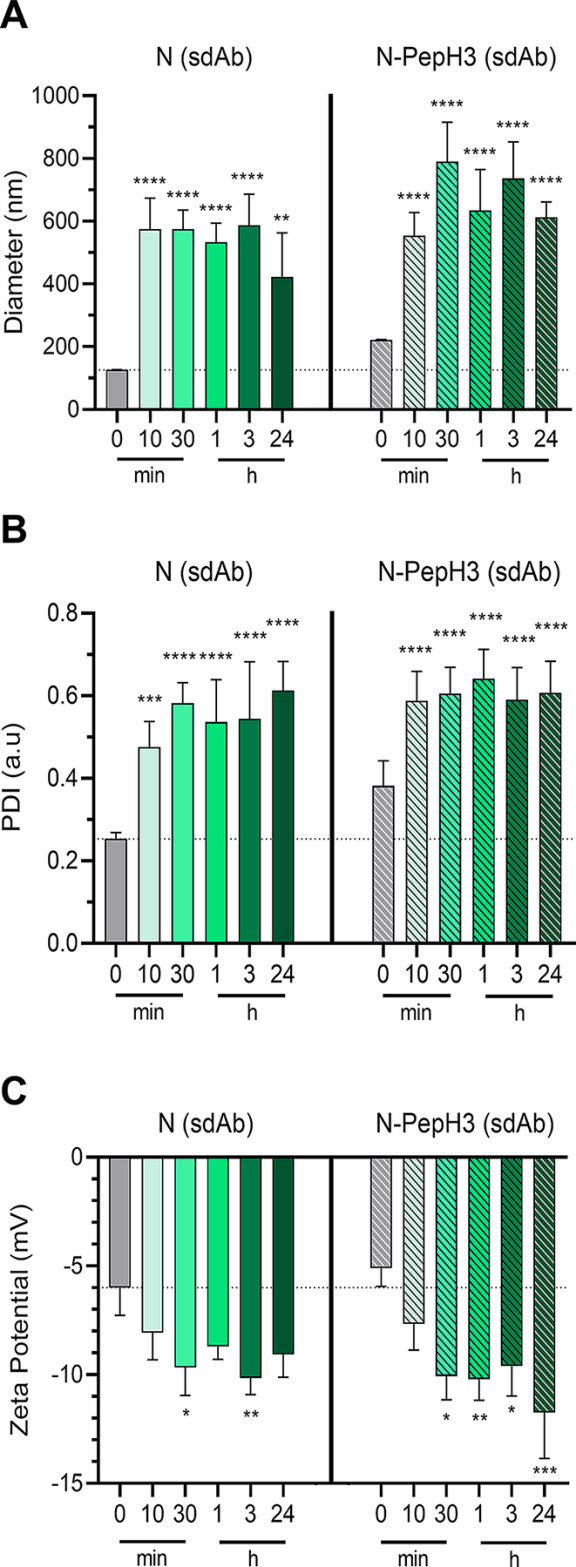
Study of the protein corona of non-tagged and PepH3-tagged niosomes with sdAb cargo. **A** Mean diameter of sdAb loaded NPs after 0, 10 and 30 min, 1, 3 and 24 h diluted in serum. Values presented are means ± SD. Statistical analysis: one-way ANOVA followed by Bonferroni post-test; **p < 0.01, ****p < 0.0001 compared to the N group at 0 min treatments; n = 3–8. **B** The polydispersity index of sdAb encapsulated NPs after 0 min, 10 and 30 min, 1, 3 and 24 h incubated in serum. Values presented are means ± SD. Statistical analysis: one-way ANOVA followed by Bonferroni post-test; ***p < 0.001, ****p < 0.0001 compared to the N group at 0 min treatments; n = 3–9. **C** The zeta potential of sdAb encapsulated NPs after 0 min, 10 and 30 min, 1, 3 and 24 h in serum condition. Values presented are means ± SD. Statistical analysis: one-way ANOVA followed by Bonferroni post-test; *p < 0.05, **p < 0.01, ***p < 0.001 compared to the N group at 0 min treatments; n = 3–8. N: non-tagged NP; N-PepH3: PepH3-tagged NP; sdAb: single-domain antibody

The time dependent changes of protein corona formation in serum condition were monitored by measuring the size, PDI and zeta potential of NPs at different time points (Fig. 3). After 10 min of 50% dilution in human plasma, the size of untagged NPs increased 5.4-fold, while the increase in N-PepH3 group was 2.5-fold (Fig. 3A), followed by an increase in PDI values (Fig. 3B). After 10 min, the initial, rapidly formed protein corona did not change significantly over the monitored 24 h. Based on zeta potential measurements, this protein corona resulted in a more negative surface charge both in the N and N-PepH3 groups (Fig. 3C).

### Effect of PepH3 and the nanoparticles on the viability of brain endothelial cells

The effect of nanovesicles on cell viability was studied both on primary RBECs and human HBEC-5i brain endothelial cells. The cellular response of RBECs to PepH3 peptide alone (Supplementary Fig. S2) and to the tagged NPs (Supplementary Fig. S3) was monitored for 24 h by real-time impedance measurements. Neither the peptide treatment nor the incubation of RBECs with TR-BSA-filled NPs (Supplementary Fig. S3A-B), or sdAb encapsulated in N and N-PepH3 NPs (Supplementary Fig. S3C-D) reduced the impedance of the cell layers, reflecting good cell viability. The effect of different NPs on the viability of HBEC-5i brain endothelial cells was studied by a colorimetric endpoint assay. The sdAb loaded niosomes did not change the viability of HBEC-5i up to 100 µg/mL concentration at the 24h time-point (Supplementary Fig. S4).

### Cellular internalization of PepH3 peptide

**Fig. 4:**
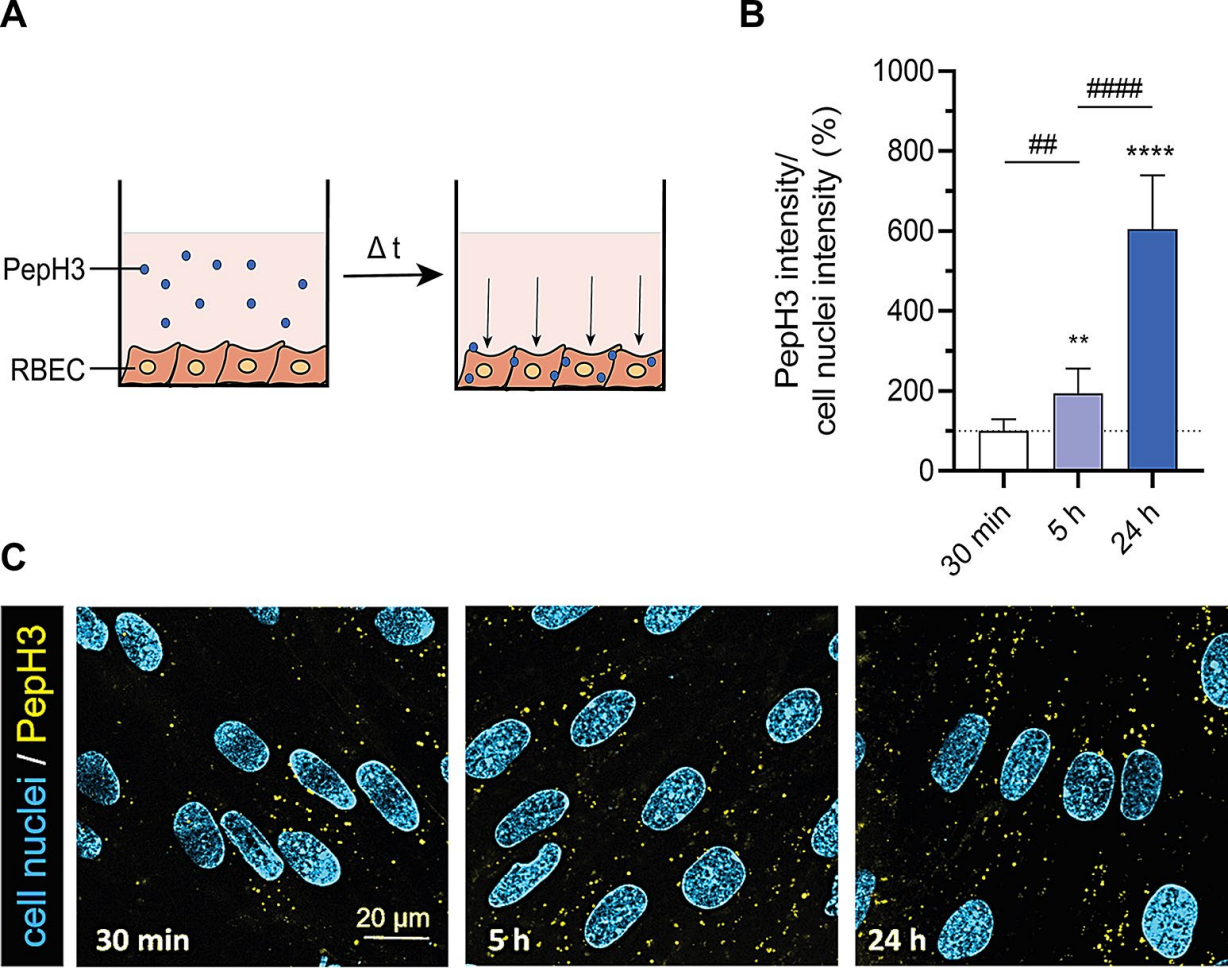
Live cell visualization of the uptake of Quasar 570 labelled PepH3 peptide (100 nM) in RBECs after 30 min, 5  and 24 h treatments. **A** Schematic picture of cellular uptake of PepH3 peptide. **B** Cellular uptake visualization of PepH3 peptide inside the living RBECs. Yellow: PepH3 peptide; cyan: cell nuclei. Scale bar: 20 μm. **C** Image analysis of cellular internalization of PepH3 peptide with normalization of nuclear intensity. Values presented are means ± SD. Statistical analysis: one-way ANOVA followed by Bonferroni post-test; **p < 0.01, ****p < 0.0001 compared to 30 min group; ##*p* < 0.01, ####*p* < 0.0001 compared to 5 h group; n = 10–25

The cellular uptake measurements of the PepH3 peptide are schematically shown in Fig. 4A. The selection of time-points for PepH3 peptide was based on our previous studies [11]. In brain endothelial cells the fluorescent signal of Quasar570 labelled PepH3 was visible after 30 min incubation by confocal microscopy (Fig. 4B-C, Supplementary Fig. S5A), reflecting rapid cellular internalization of the peptide. After 5 and 24 h incubations, time-dependent elevation in the cellular uptake of PepH3 was seen in living cells (Fig. 4B-C, Supplementary Fig. S5A). The peptide internalization was 1.9-fold after 5 h treatment, while after 24 h incubation it was 6-fold higher compared to the 30 min group (Fig. 4B-C).

### Cellular internalization of the cargo of PepH3 tagged and non-tagged nanoparticles

**Fig. 5:**
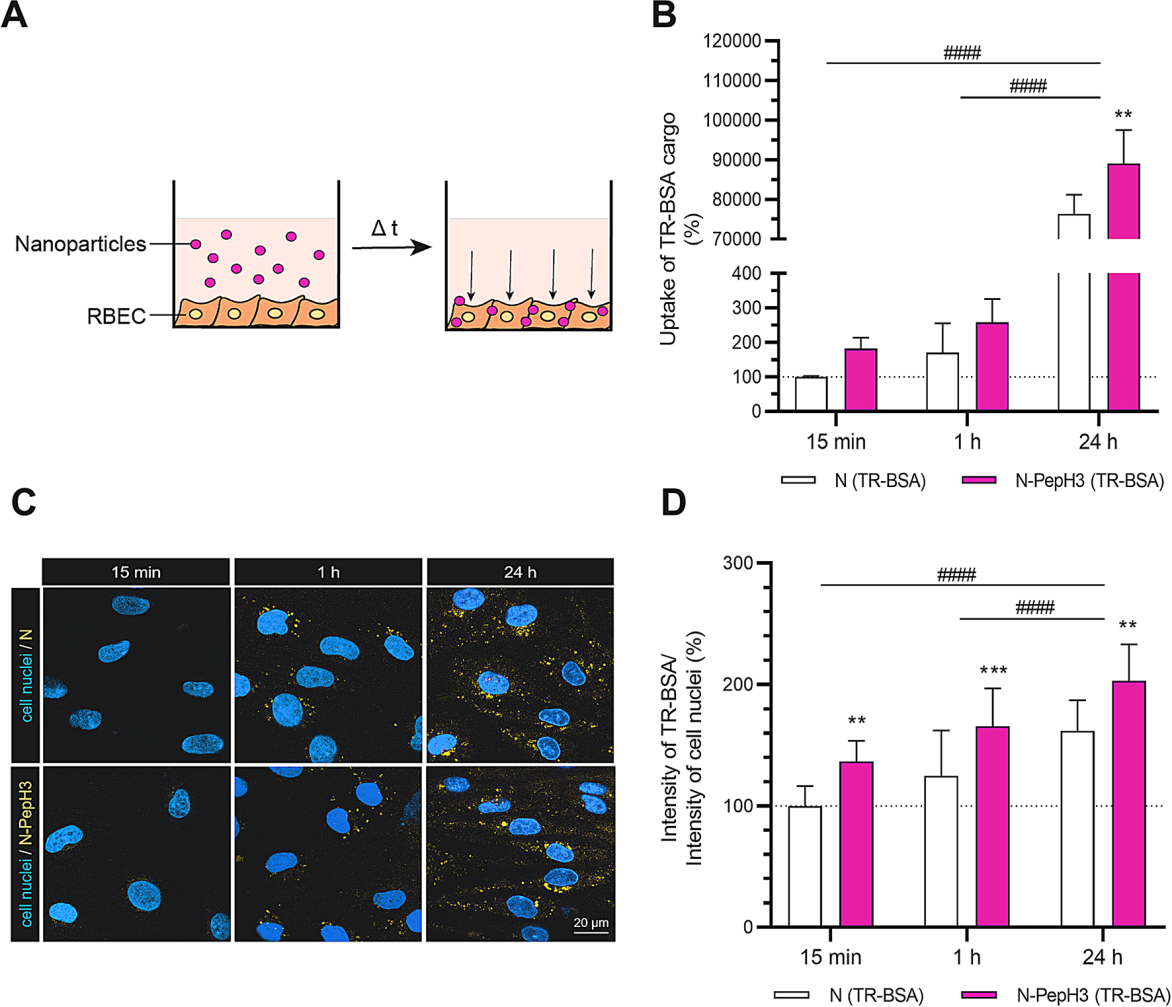
Cellular internalization of Texas Red BSA encapsulated NPs (1 mg/mL) into rat brain endothelial cells after 15 min, 1 and 24 h NPs treatments. **A** Schematic drawing of cellular uptake of NPs. **B** Quantification of cellular internalization of Texas Red BSA encapsulated NPs taken up by rat brain endothelial cells after 15 min, 1 and 24 h NPs treatments. Values presented are means ± SD and are given as a percentage of the control group at both time points. Statistical analysis: two-way ANOVA followed by Bonferroni post-test; **p < 0.01 compared to N group; ####*p* < 0.0001 compared to 24 h group; n = 3–4. **C** Representative confocal microscopy images of cellular uptake. Yellow: non-tagged or PepH3 tagged NPs; blue: cell nuclei. Scale bar: 20 μm. **D** Evaluation of the fluorescence intensity of TR-BSA cargo inside the living cell with normalization of nuclear intensity. Values presented are means ± SD. Statistical analysis: two-way ANOVA followed by Bonferroni post-test; **p < 0.01, ***p < 0.001 compared to N group; ####*p* < 0.0001 compared to 15 min group; n = 10–15. N: non-tagged NP; N-PepH3: PepH3-tagged NP; TR-BSA: Texas Red-labelled bovine serum albumin

After 15 min, 1 and 24 h incubation times, visualization and quantification of the uptake of TR-BSA cargo of non-tagged and PepH3- tagged NPs was studied in RBECs (Fig. 5). The entry of encapsulated TR-BSA cargo into living RBECs was significantly increased with time (15 min, 1 and 24 h incubation with NPs) as studied by confocal microscopy (Fig. 5C). The image analysis revealed that PepH3 peptide, as a BBB shuttle on the surface of the niosomes successfully increased the cellular internalization of TR-BSA cargo at each time point, compared to the non-tagged NP treated group (Fig. 5D). The fluorescent intensity of TR-BSA in the case of N-PepH3 treated group was 1.25-fold higher compared to N group (N-PepH3: 203%, N: 162%) after 24 h incubation (Fig. 5D). To quantify the cellular internalization of TR-BSA cargo in RBECs, cells were treated with TR-BSA loaded N and N-PepH3 and the uptake was measured by spectrofluorometer (Fig. 5B). In concordance with the results of image analyses (Fig. 5D), the uptake of NP cargo into the cells was elevated in a time-dependent way (Fig. 5B).

### The cellular uptake mechanism of the nanoparticle cargo

**Fig. 6:**
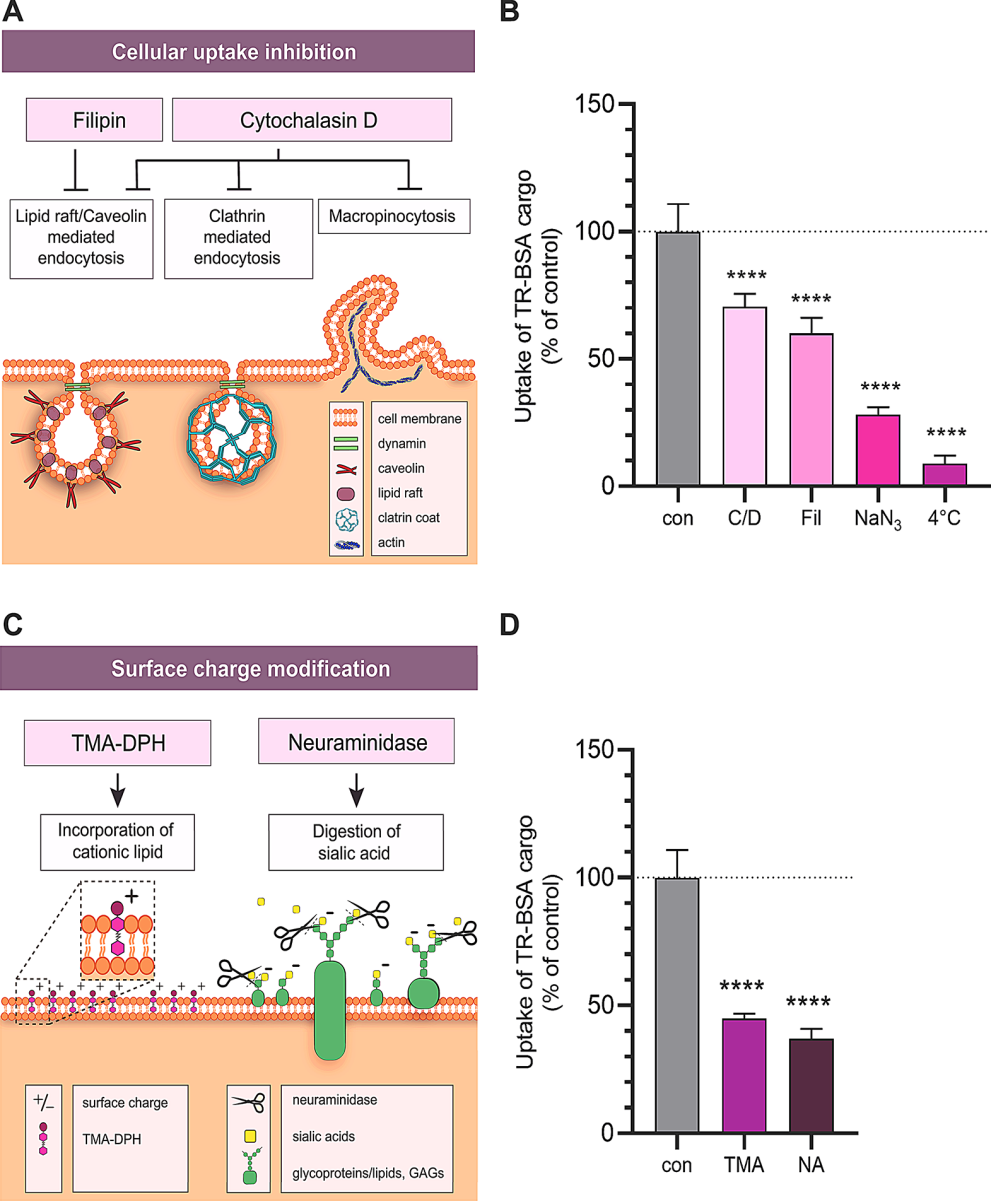
**A** Schematic illustration of the inhibition of cellular uptake mechanisms. **B** The effect of endocytic inhibitor cytochalasin D (C/D; 0.125 µg/mL) and filipin (Fil; 5 µg/mL) or metabolic inhibitor sodium azide (NaN_3_; 1 mg/mL), and incubation at 4ºC on the cellular uptake of TR-BSA loaded N-PepH3. Control (con): N-PepH3 treatment alone at 37 ºC without any inhibitors. Values presented are means ± SD and are given as a percentage of the control group. Statistical analysis: one-way ANOVA followed by Bonferroni post-test; ****p < 0.0001 compared to control group; n = 6. **C** The effect of the modification of glycocalyx with neuraminidase (NA; 1 U/mL) and TMA-DPH (TMA; 30 mM) on the cellular uptake of TR-BSA loaded N-PepH3 (37ºC, 24 h). Control: N-PepH3 treatment alone at 37 ºC without charge modification reagents. Values presented are means ± SD and are given as a percentage of the control group. Statistical analysis: one-way ANOVA followed by Bonferroni post-test; ****p < 0.0001 compared to control group

To reveal the mechanism of cellular uptake of NPs cargo, we pre-treated the RBECs with lipid raft/caveolin-dependent endocytosis inhibitor filipin (5 µg/mL, 15 min), or actin polymerization blocking agent cytochalasin D (0.125 µg/mL, 1 h) that inhibits all major endocytic routes (Fig. 6A-B, Supplementary Fig. S6). The uptake of cargo was significantly reduced when the cells were treated with filipin or cytochalasin D compared to the control group suggesting that the cellular internalization of N-PepH3 was partially mediated by endocytosis in RBECs. In order to investigate if ATP synthesis is contributing to the cellular uptake of NPs, we used metabolic inhibitor sodium azide and also tested the uptake of NPs at 4 °C. Sodium azide or incubation at 4 °C resulted in significantly less uptake of TR-BSA cargo into the cells compared to the control group, indicating energy dependent cellular internalization of N-PepH3 (Fig. 6A-B). To verify the role of the negatively charged glycocalyx in the NP uptake process, we modified the surface charge of RBECs by removing the negative sialic acid residues from the glycocalyx by digestion with neuraminidase enzyme, or treating the cells with cationic lipid TMA-DPH (Fig. 6C-D). Due to these treatments, the cellular uptake of the cationic PepH3-tagged NPs was significantly decreased compared to the control groups (Fig. 6C-D). Unmodified NPs also entered to brain endothelial cells in an energy dependent way, which was partially mediated by endocytosis (Supplementary Fig. S6A). However, the cellular uptake of the untargeted NPs was inhibited by TMA-DPH and neuraminidase treatments less (Supplementary Fig. S6B) compared to the targeted NPs (Fig. 6D).

### Colocalization of the cargo of the nanoparticles with Endoplasmic reticulum, golgi and lysosomes

**Fig. 7:**
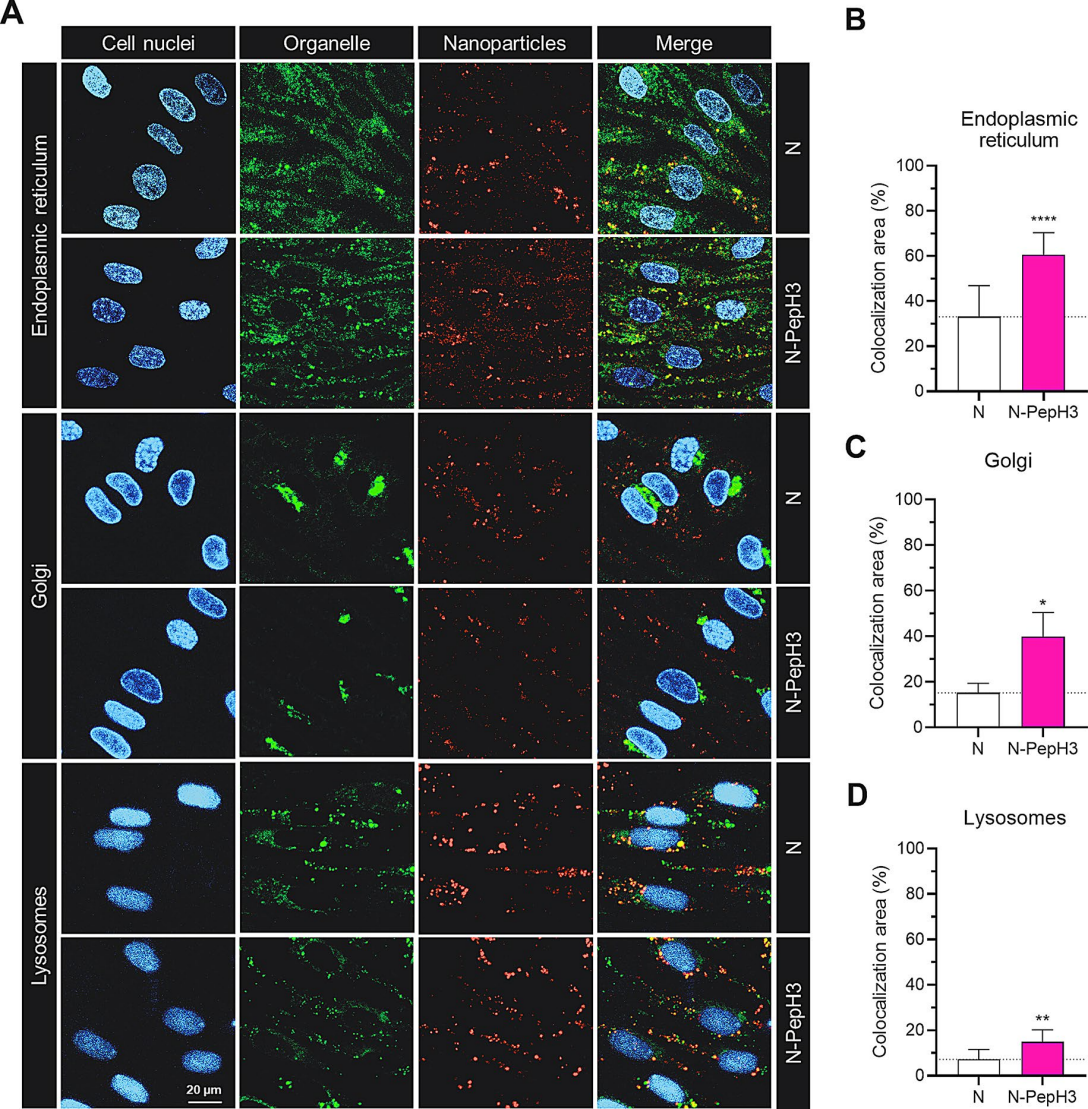
Visualization of the NPs in intracellular compartments. **A** Colocalization of TR-BSA cargo with endoplasmic reticulum, Golgi or lysosomes in cultures of RBEC cells after 24 h incubation of NPs (1 mg/mL). Cyan: cell nuclei; green: endoplasmic reticulum, Golgi apparatus or lysosomes; red: TR-BSA cargo of non-tagged (N) or PepH3 tagged (N-PepH3) nanovesicles; Scale bar: 20 μm. Image analyses to determine the colocalization area of NP cargo with **B** Endoplasmic reticulum, **C** Golgi apparatus or **D** Lysosomes. Values presented are means ± SD and are given as a percentage of colocalization area. Statistical analysis: unpaired t-test; *p < 0.05, **p < 0.01, ****p < 0.0001 compared to non-tagged group; n = 4–10

To investigate the intracellular trafficking of NPs inside the cells the co-localization of TR-BSA loaded NPs with cell organelles such as ER, Golgi and lysosomes were visualized by confocal microscopy (Fig. 7A). The pixel-based image analysis correlated with object recognition showed that the biggest colocalization area of NPs cargo (N: 33%; N-PepH3: 67%) was determined with ER (Fig. 7B). The second highest colocalization level (N: 15%; N-PepH3: 40%) was detected with Golgi (Fig. 7C). A limited amount of cargo was colocalized (N: 7%; N-PepH3: 15%) with lysosomes (Fig. 7D). The rate of the colocalization was higher in PepH3 tagged cells compared to the non-tagged NP treated group.

### Permeability of PepH3 peptide and PepH3-tagged nanoparticles across the rat and human BBB models

**Fig. 8:**
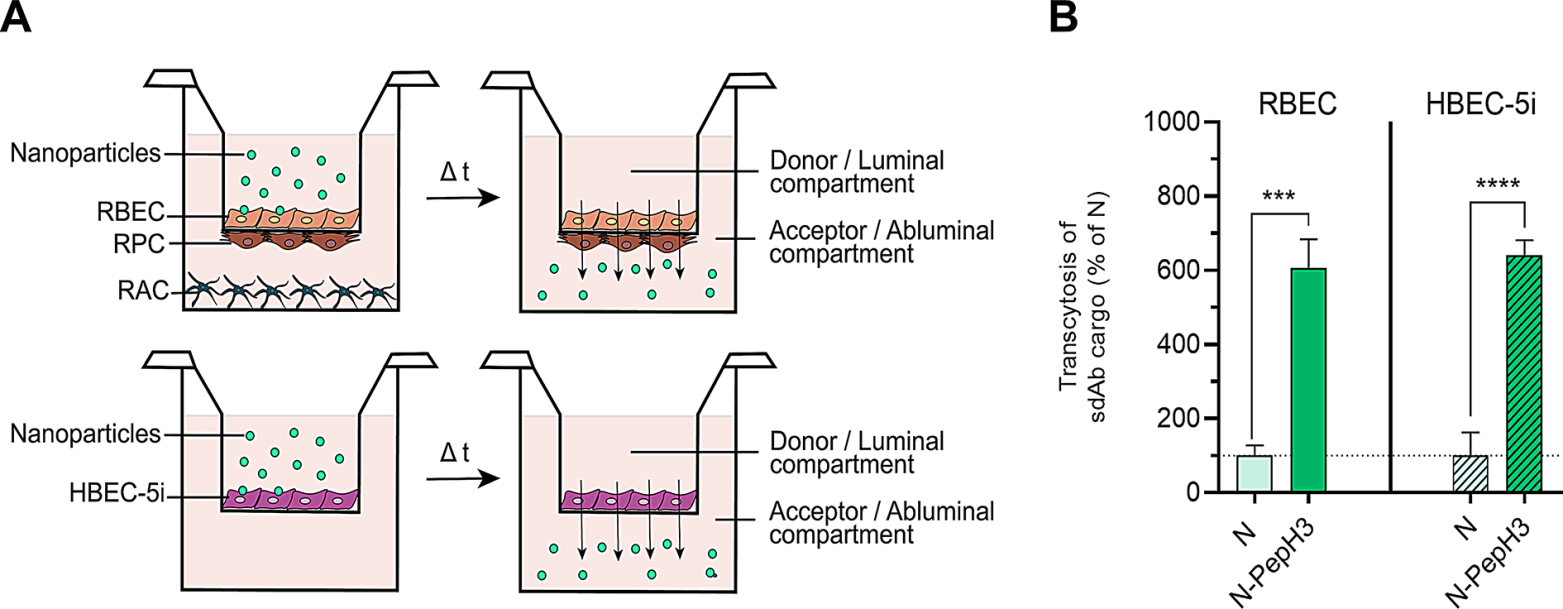
Translocation of the NPs across BBB models. **A** Schematic drawing of the permeability assay for NPs on the primary rat and human BBB culture models. **B** Transcytosis of sdAb encapsulated in non-tagged (N, 1 and 0.1 mg/mL) and PepH3-tagged NPs (N-PepH3, 1 and 0.1 mg/mL) across the rat and human BBB models after 24 h of NPs incubation. Values presented are means ± SD. Statistical analysis: unpaired t-test; *** *p* < 0.001, **** *p* < 0.0001 compared to N groups; n = 4–6. RBEC: rat brain endothelial cells; RPC: rat brain pericytes; RAC: rat astroglial cells; HBEC-5i: human brain endothelial cell line

The penetration of PepH3 peptide alone across the BBB was tested on a rat primary cell-based co-culture model (Fig. 8A). The P_app_ of the PepH3 peptide (10 nM, 30 min) was 2.5 × 10^− 6^ cm/s, which was 71 times higher compared to the transcellular BBB marker molecule albumin, indicating high penetration of PepH3 across the BBB (Supplementary Fig. S7).

The translocation of sdAb encapsulated in NPs was investigated on both the rat co-culture BBB model and the human monoculture model of HBEC-5i endothelial cell line (Fig. 8A-B). To verify the integrity of the BBB culture models, the penetration of water-soluble paracellular permeability markers with different size (sodium fluorescein, 376 Da; fluorescein isothiocyanate-dextran, 4 kDa) and transcellular marker albumin (Evans blue-albumin, 67 kDa) across the models were measured. The low permeability values of the reference markers indicated a tight barrier in both models of the BBB (Supplementary Fig. S8, S9). The sdAb cargo of the NPs was detected by Western blot both in the apical and basolateral compartments after 24 h permeability measurements. PepH3-tagged NPs increased significantly and similarly the translocation of the sdAb cargo (Fig. 8B) across the rat BBB (6.1-fold) and the human HBEC-5i models (6.4-fold).

## Discussion

Most of the drug candidates developed for Alzheimer's disease have limited access to their brain targets because the BBB restricts the entry of therapeutic compounds into the CNS [30]. To increase drug delivery and to avoid degradation in the circulation, a possible approach for Alzheimer's disease therapies could be to encapsulate the therapeutic molecules including anti-Aβ mAbs into nanocarriers [3, 4].

The BBB, that is present at the level of the brain capillaries, is mainly composed of cerebral endothelial cells [30]. Over the past two decades, natural or synthetic oligopeptides have been extensively studied to facilitate the passage of drugs across cell membranes and to act as carrier molecules to increase the accumulation of drugs in target cells. These cell-penetrating peptides typically consists of short sequences of 5–30 amino acids [31, 32] However, not all cell-penetrating peptides are able to deliver agents into the brain [12], a significant portion of them remains internalized within cells or fail to sufficiently cross the BBB due to protection systems, such as efflux pump activity. Peptides that facilitate the passage of drug molecules and nanocarrier systems across the BBB are referred to BBBpS. Drug-conjugated BBBpS or BBB-specific biomolecules tagged nanocarriers enhance drug delivery across the BBB by utilizing physiological transport pathways, including carrier-mediated, receptor-mediated or adsorptive-mediated transcytosis [9, 10].

One of the widely studied BBBpS or targeting ligand for NPs is the tripeptide glutathione, which successfully elevates the drug penetration across the BBB based on cell culture, animal and clinical studies [4, 33, 34]. In accordance with these results, our research group previously demonstrated that glutathione, its combination with alanine (N-A-GSH) [6, 7, 35] and the triple functionalization of NPs with glutathione, ascorbic acid and leucine (N-AGL) [8] significantly increased the cellular internalization and penetration of nanocarriers across well characterized co-culture models of the BBB. In addition to glutathione, numerous BBBpSs as targeting ligands of NPs, such as HIV-1 Tat, rabies virus glycoprotein 29, penetratin, transportan, polyarginines, ApoE and angiopep-2 elevated the brain penetration of therapeutic cargos in both in vitro and in vivo studies [3, 10, 36–38].

In the present study, we selected the cationic PepH3 as a shuttle ligand, which is a peptide fragment of Dengue virus capsid protein-2, to enhance the NP penetration across the BBB. Previously, PepH3 peptide alone had a high cellular uptake and permeability across the HBEC-5i and bEnd.3 endothelial cell culture models of the BBB [12, 24, 39]. In accordance with these results, we confirmed that the Quasar 570 labelled PepH3 alone had no negative effects on the viability of brain endothelial cells (Supplementary Fig. S2), was rapidly taken up by RBECs (Fig. 4), and translocated across the primary cell-based co-culture model of the BBB (Supplementary Fig. S7).

### Characteristics of the nanocarriers

We designed PepH3-tagged vesicular NPs to cross the BBB for the experiments. We prepared PepH3-functionalized NPs with fluorescent TR-BSA as a model cargo or sdAb as a therapeutic biomolecule (Fig. 1A) that binds to the oligomeric form of aggregated Aβ peptide and blocks the further aggregation of Aβ [4, 40]. The average diameter of non-tagged and N-PepH3 NPs (Fig. 1B) was in the size range sufficient for brain drug delivery [2, 41]. For brain-targeted NPs the importance of a low (< 0.3) PDI is considered important to enhance their ability to cross the BBB and ensure uniform drug delivery [42], which depend on hydrodynamic size and shape [43]. However, other studies suggest that formulations with PDI ≤ 0.5 are still suitable for such applications, especially if accompanied by other favorable characteristics like appropriate particle size (< 200 nm) and surface modifications to facilitate BBB penetration [44]. The PDI values of our NPs are in this range indicating good size distribution (Fig. 1B).

The cell-penetrating peptide PepH3 contains seven amino acids (AGILKRW), including the cationic residues arginine and lysine, which give two positive charges and contribute to the cationic nature of the peptide [11]. This positive charge is a critical feature that enhances the peptide’s ability to interact with the negatively charged brain endothelial cell surface and contributes to its cell-penetrating properties [10, 45]. In accordance with the cationic characteristics of PepH3, the zeta potential of the PepH3-targeted nanovesicles (Fig. 1B) was significantly less negative as compared to non-targeted NPs. The cationic PepH3, as a targeting ligand on the surface of the nanocarriers, increases the zeta potential by ~ 2 mV in a statistically significant way (Supplementary Fig. S1) that can also support the better uptake of NPs by brain endothelial cells [46].

The encapsulation efficiencies of the large molecular weight cargo TR-BSA in non-tagged and PepH3-tagged NPs was effective for its size and similar to previous data [8, 47]. In the case of the smaller model therapeutic cargo sdAb higher encapsulation was measured (Fig. 1B). The physico-chemical properties of NPs were stable both in PBS and in cell culture medium at pH 5.0 and 7.4, except in the case of non-tagged NPs, where the diameter and PDI increased after 24 h incubation in acidic condition (Fig. 2) indicating lower stability caused by the lack of PEGylation [48]. On the other hand, the cationic PepH3-tagged NPs resulted in higher diameter in all conditions and increased the adsorption of negatively charged plasma proteins compared to the non-tagged NPs (Fig. 3). The rapidly formed protein corona on the NPs may define a biological response and influence the cellular uptake and penetration of NPs across the BBB [49–52].

### The cellular uptake mechanism of PepH3-tagged NPs

Supporting our hypothesis, PepH3 as a tag on the surface successfully and time-dependently increased the cellular internalization of NPs as compared to non-tagged NPs based on sensitive spectrofluorometer measurement and semi-quantitative confocal microscopy (Fig. 5). To elucidate the cellular uptake mechanisms of PepH3-tagged NPs, we applied sodium azide and low-temperature to inhibit mitochondrial ATP production and active processes in cells. Both conditions reduced the cellular entry of the NPs reflecting an energy-dependent internalization process, similarly to our previous studies with dual- and triple-targeted NPs [7, 8, 35].

Many studies have reported that the major pathways of the uptake of BBBpS and BBBpS-tagged NPs could be macropinocytosis, clathrin- or caveolae-dependent endocytosis, or they might exploit multiple entry routes simultaneously [53, 54]. To determine that the cellular uptake mechanisms involve endocytosis, we pre-treated brain endothelial cells with cytochalasin D and filipin [35]. Cytochalasin D by F-actin depolymerization non-selectively inhibits endocytosis and microfilament formation for micropinocytosis [55]. Filipin is a blocker of the lipid raft/caveolin-dependent endocytic pathway by acute cholesterol depletion of the plasma membrane [56]. Treatment with cytochalasin D and filipin successfully decreased the internalization of the cargo (Fig. 6), indicating that the entry of PepH3-tagged NPs into the cells is, at least partially, mediated by endocytosis as it was previously described in the case of N-A-GSH and N-AGL functionalized nanocarriers [7, 8, 35].

The surface charge of NPs may influence their binding and uptake in brain endothelial cells that exhibit a highly negative surface charge [47]. Cationic BBBpSs, like Tat or SynB as targeting ligands are able to bind to the negatively charged chains of the glycocalyx on the luminal side of brain endothelial cells and initiate endocytosis [57]. To investigate the contribution of cell surface charge to the enhanced uptake of cationic PepH3-tagged NPs, we made the charge of brain endothelial cells more positive using the cationic lipid TMA-DPH and neuraminidase to remove sialic acid residues (Fig. 6C). TMA-DPH reduced the uptake of NPs to about half, while neuraminidase pre-treatment decreased to one third the amount of cargo internalization (Fig. 6D). These data proved that the brain endothelial cell surface electrostatic barrier [45] significantly influences the uptake of the cationic PepH3-tagged NPs.

We also investigated the intracellular localization of NPs with the main endo-lysosomal compartments, such as ER, Golgi and lysosomes (Fig. 7). The largest colocalized area of NP cargo was determined with the ER, and the second-highest accumulation level was detected with the Golgi. Only a limited amount of cargo showed intracellular localization in lysosomes, indicating that most of the cargo avoided cellular degradation. In brain endothelial cells several elements of the vesicular transport machinery have been identified, including clathrin-coated pits, caveolae, and macropinocytic vesicles [58, 59]. The caveolae-mediated uptake has the capability to avoid the lysosomes and deliver NPs to the Golgi apparatus and ER by retrograde trafficking [54] and this pathway is also active in brain endothelial cells [59, 60].

BBB transcytosis consists of three key steps: endocytosis, intracellular vesicular trafficking, and exocytosis. In a previous study, it was determined that the PepH3 peptide was successfully blocked by brefeldin A treatment, which is an inhibitor of GTPases that disrupts vesicle formation and trafficking between the ER and the Golgi apparatus, indicating an endosomal dependent trafficking [44]. Our results have shown that PepH3-tagged NPs were internalized via electrostatic interaction with negatively charged glycocalyx of brain endothelial cells, leading to an energy dependent endocytic mechanism. Endocytosis occurs through formation of caveolin pits, followed by intracellular trafficking dependent of ER and Golgi. The low colocalization with lysosomes and high permeability across the BBB model suggest low degradation and successful exocytosis to the abluminal side. Overall, we suggest in the present manuscript that the permeability across the BBB and low colocalization with lysosomes indicate a transcytosis mechanism that avoids degradation in the lysosomes, although we have not investigated lysosome escape mechanisms, because only a very small fraction of PepH3-tagged NPs is entrapped/degraded in the lysosomes.

### Permeability of PepH3 peptide and PepH3-tagged nanoparticles across the rat and human blood-brain barrier model

The transport of large molecules such as proteins, peptides, and NPs are primarily limited to transcellular routes involving endo- and transcytosis. Vesicular trafficking through the BBB can occur via two major mechanisms: receptor- and adsorptive-mediated transcytosis [61]. In receptor-mediated transcytosis, the binding of macromolecular ligands, e.g. transferrin, insulin, leptin, or apolipoprotein E, to specific receptors on the cell surface initiates the endocytic event. In adsorptive-mediated transcytosis, a positive charge on the molecule or NP facilitates interaction with the anionic brain endothelial surface, which electrostatically triggers transcytosis [57, 61]. Adsorptive-mediated transcytosis exhibits low binding affinity, but high binding capacity, resulting in similar transcytotic efficiency to the receptor-mediated pathway [57]. Based on our previous results, the penetration of the cationic PepH3 peptide was shown to be independent of receptors and associated with adsorptive-mediated endocytosis [12, 24]. Adsorptive-mediated transcytosis is a process that is concentration, time and energy dependent. However, adsorptive-mediated transcytosis is saturated at higher concentration than the receptor-mediated one, which in principle will allow higher amount of drug in the brain [57]. In addition, adsorptive-mediated transcytosis is not dependent on the overexpression of specific receptors at the BBB and is not limited by competition with natural ligands. In our previous study, we compared the in vivo BBB permeability of PepH3 with other BBBpS peptides, such as TAT, SynB, angiopep-2, dNP2, TP10, miniAp-4, and we confirmed that the PepH3 peptide has shown similar biodistribution profile with SynB, dNP2, and improved BBB crossing capacity compared to angiopep-2 and TP-10 [11]. Moreover, we have reported on the strong evidence that PepH3 returns to blood circulation to be excreted, which is important to avoid accumulation in the brain and possible toxic effects [11].

The present work focused on the development of new nanocarriers with PepH3 targeting to deliver therapeutic biomolecules (sdAb) across the BBB. For this purpose, we used two separate BBB models derived from different species, the rat BBB co-culture model consisting of primary RBECs, pericytes and astrocytes and human cerebral microvascular endothelial HBEC-5i cell line. We found that the permeability of PepH3-tagged NPs showed more than 6-fold higher penetration through both the primary rat co-culture and human HBEC5i monoculture models in comparison with non-tagged NPs (Fig. 8B). We hypothesize, that the presence of PepH3 peptide on the surface of NPs may enhance the penetration of sdAb cargo by the adsorptive-mediated transcytosis pathway, similarly to our previous studies on the peptide alone [12, 24].

It is a limitation of the present work, that no in vivo data are provided. In concordance with the “3R” principles to refine, reduce and replace the number of animal experiments, our aim was to develop an innovative tagged NP that crosses cell culture models of the BBB and acts as a shuttle peptide for the encapsulated sdAb recognizing Aβ oligomers. The results showed improvement in the transport of nanocarriers across both rat and human BBB models, when using PepH3-tagged NPs vs. non-tagged. The in vitro results from the present study can provide a strong foundation for future in vivo investigations into therapeutic efficacy, brain uptake and biodistribution studies. However, before progressing to in vivo studies, it is essential to optimize the preparation of nanocarriers to enable large-scale production, safety and efficacy which was not in the scope of the present manuscript and will be addressed in future work.

## Conclusion

In summary, we have shown that PepH3 peptide, as a tagging ligand of nanocarriers, is not only able to enhance the cellular uptake of the fluorescent cargo of NPs into brain endothelial cells, but also the transcytosis of sdAb as a potential therapeutic molecule across the BBB. Our results support that PepH3 peptide conjugated to nanocarriers efficiently increase the delivery of large cargos, such as antibodies or other therapeutic biomolecules across the BBB.

## Electronic supplementary material

Below is the link to the electronic supplementary material.

Supplementary Material 1: Figure S1. Zeta potential of non-tagged and PepH3-tagged niosomes with albumin or single-domain antibody cargo. Figure S2. The effect of PepH3 (1.875-120 μM) on the viability of RBECs after 24 h incubation. Figure S3. The effect of NPs (0.1-3 mg/mL) on the viability of RBECs. Figure S4. The effect of sdAb loaded non-tagged and PepH3-tagged NPs on the viability of HBEC-5i cell line. Figure S5. Time-dependent cellular uptake of the PepH3 peptide and NPs where the fluorescence intensity values were normalized to cell nuclei number. Figure S6. Cellular uptake mechanisms of the non-targeted TR-BSA loaded nanoparticles. Figure S7. Permeability of Quasar 570 labelled PepH3 peptide (1.3 kDa, 10 nM, 30 min) and the BBB marker molecule, albumin (67 kDa) across the rat BBB model. Figure S8. Permeability of transcellular BBB marker, EBA and the paracellular BBB marker, SF across the rat BBB model after 30 min and 24 h incubation. Figure S9. Permeability of the BBB marker FD4 (4 kDa) across the human BBB model after 2 h incubation of NPs.

## Data Availability

The dataset used and/or analysed during the current study are available from the corresponding author on reasonable request.

## Abbreviations

AD: Alzheimer’s disease
Aβ: Amyloid-beta
BBB: Blood-brain barrier
BBBpS: Blood-brain barrier peptide shuttle
bFGF: Basic fibroblast growth factor
BSA: Bovine serum albumin
C/D: Cytochalasin D
CNS: Central nervous system
cPT-cAMP: 8-(4-chlorophenylthio) adenosine 3’,5’-cyclic monophosphate
DMEM: Dulbecco’s modified Eagle medium
EBA: Evans blue-labeled albumin
EE%: Encapsulation efficiency percentage
ER: Endoplasmic reticulum
FBS: Fetal bovine serum
FD4: Fluorescein-dextran 4 kDa
Fil: Filipin
HBEC-5i: Human cerebral microvascular endothelial cells
ITS: Insulin, transferrin, sodium selenite
mAbs: Monoclonal antibodies
N: Non-tagged nanoparticles
NA: Neuraminidase
NaN_3_: Sodium azide
NP: Nanoparticle
N-PepH3: PepH3-tagged nanoparticle
P_app_: Apparent permeability coefficient
PBS: Phosphate buffered saline
PDI: Polydispersity index
PDS: Plasma-derived bovine serum
PEG: Polyethyleneglycol
RAC: Primary rat brain astrocytes
RBEC: Primary rat brain endothelial cells
RPC: Primary rat brain pericytes
sdAb: Single domain antibody
SF: Sodium fluorescein
Solulan: C24 cholesterylpoly-24-oyxyethylene-ether
Span 60: Sorbitane-monostearate
TMA-DPH: 1-(4-trimethylammoniumphenyl)-6-phenyl-1,3,5-hexatriene
TR-BSA: Texas red labelled bovine serum albumin
