# Supplementary material for: PepH3-modified nanocarriers for delivery of therapeutics across the blood-brain barrier
Source: Fluids Barriers CNS. 2025 Apr 1;22:31. doi: 10.1186/s12987-025-00641-0 (PMC11959756; doi:10.1186/s12987-025-00641-0)
Supplement: Supplementary file 1 — Supplementary Material 1: Figure S1. Zeta potential of non-tagged and PepH3-tagged niosomes with albumin or single-domain antibody cargo. Figure S2. The effect of PepH3 (1.875-120 μM) on the viability of RBECs after 24 h incubation. Figure S3. The effect of NPs (0.1-3 mg/mL) on the viability of RBECs. Figure S4. The effect of sdAb loaded non-tagged and PepH3-tagged NPs on the viability of HBEC-5i cell line. Figure S5. Time-dependent cellular uptake of the PepH3 peptide and NPs where the fluorescence intensity values were normalized to cell nuclei number. Figure S6. Cellular uptake mechanisms of the non-targeted TR-BSA loaded nanoparticles. Figure S7. Permeability of Quasar 570 labelled PepH3 peptide (1.3 kDa, 10 nM, 30 min) and the BBB marker molecule, albumin (67 kDa) across the rat BBB model. Figure S8. Permeability of transcellular BBB marker, EBA and the paracellular BBB marker, SF across the rat BBB model after 30 min and 24 h incubation. Figure S9. Permeability of the BBB marker FD4 (4 kDa) across the human BBB model after 2 h incubation of NPs. [file 12987_2025_641_MOESM1_ESM.docx]

# Supplementary Material

**PepH3-modified Nanocarriers for delivery of therapeutics across the Blood-Brain Barrier**

Anikó Szecskó^#^, Mária Mészáros^#^, Beatriz Simões^#^, Marco Cavaco, Catarina Chaparro, Gergő Porkoláb, Miguel A.R.B. Castanho, Mária A. Deli, Vera Neves^*^, Szilvia Veszelka^*^


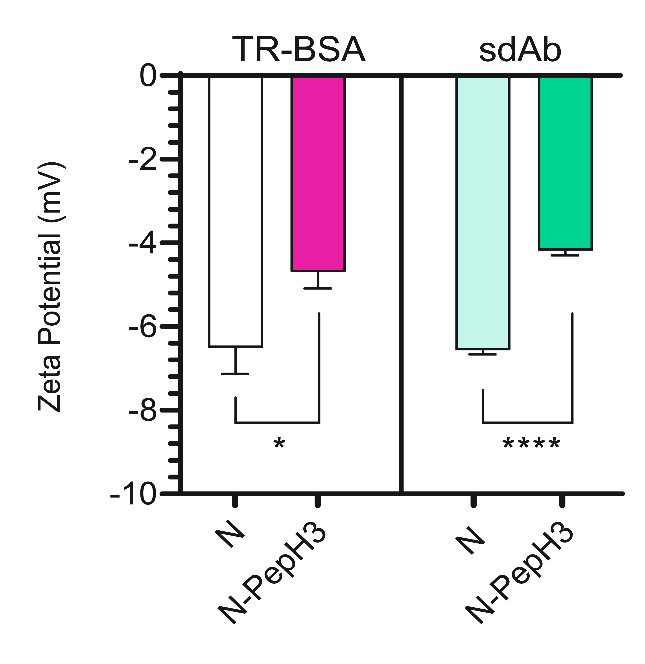


**Fig. S1** Zeta potential of non-tagged and PepH3-tagged niosomes with albumin or single-domain antibody cargo. Values presented are means ± SD; Statistical analyses: t-test; *p < 0.05, ****p < 0.0001 compared to N groups; n=3. N: non-tagged nanoparticle; N-PepH3: PepH3-tagged nanoparticle; sdAb: single-domain antibody; TR-BSA: Texas red-labelled bovine serum albumin.

**
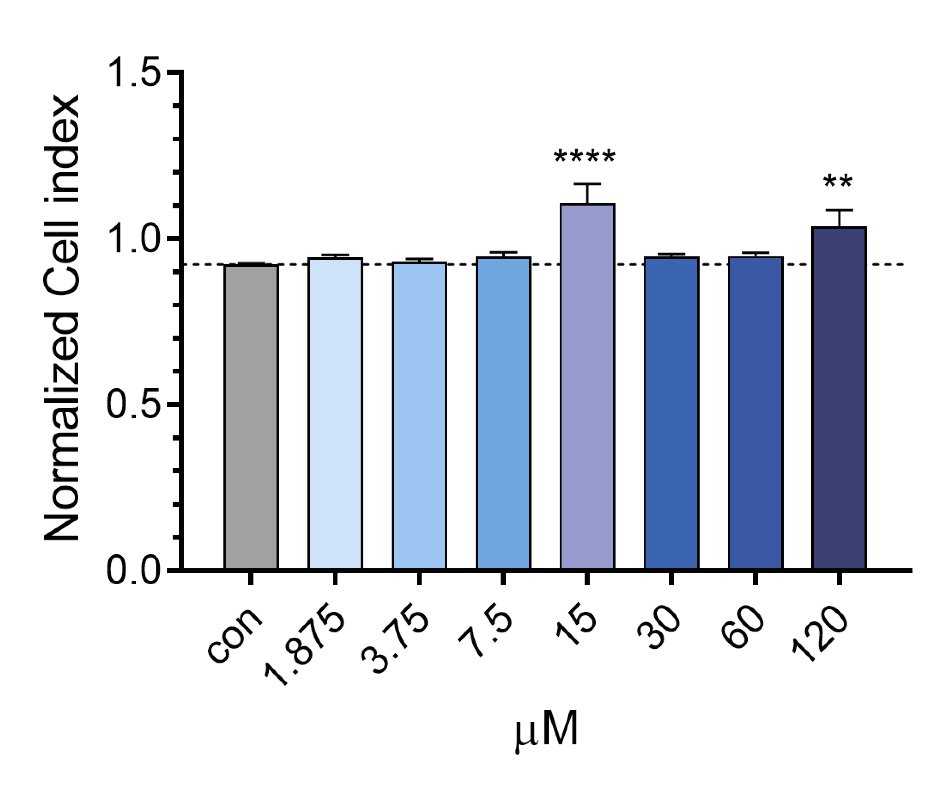
**

**Fig. S2** The effect of PepH3 (1.875-120 μM) on the viability of RBECs after 24 h incubation. Values presented are means ± SEM. Statistical analysis: one-way ANOVA followed by Bonferroni post-test; **p < 0.01, ****p < 0.0001 compared to control group; n=6-12. con: control group treated with culture medium.


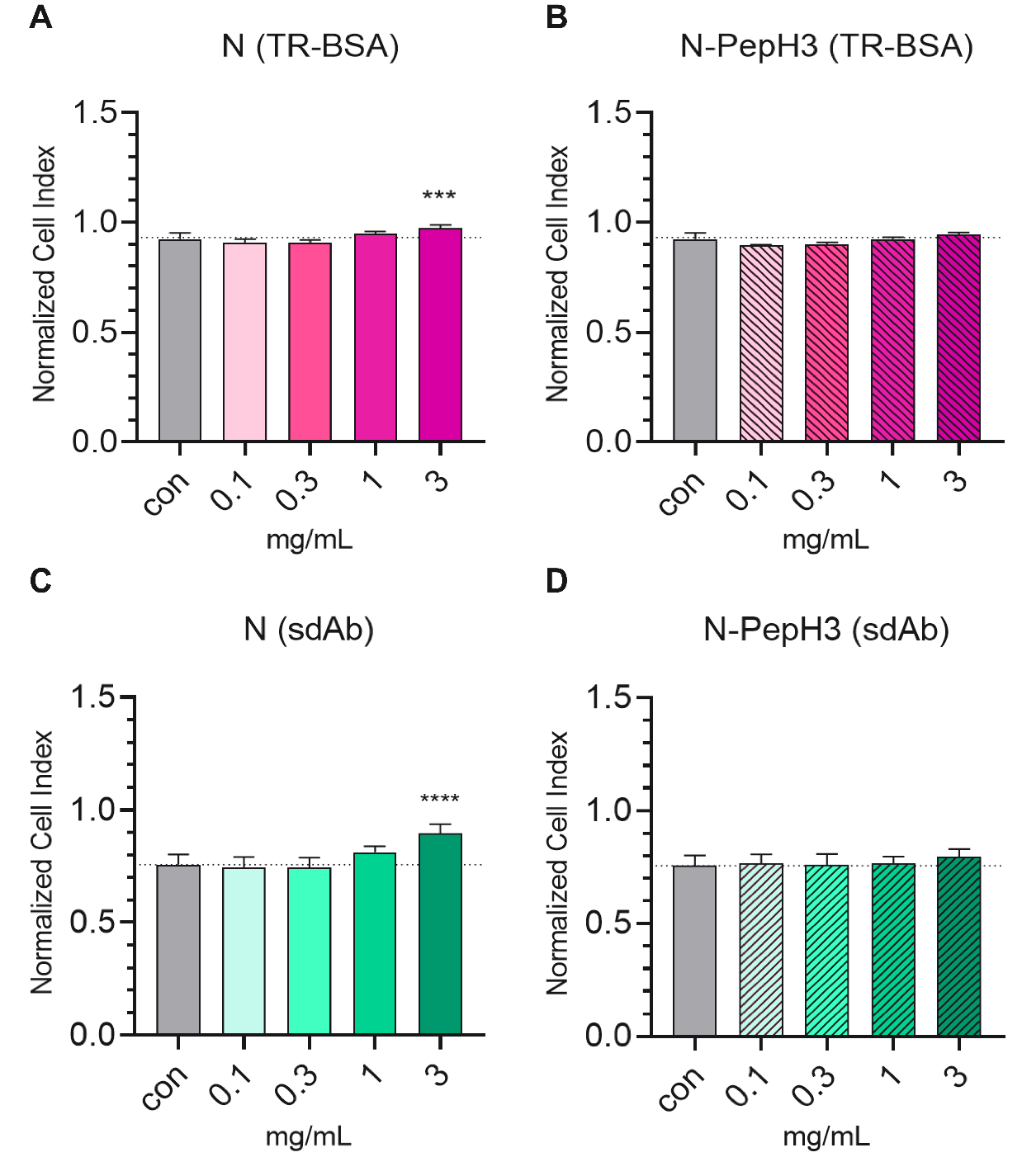


**Fig. S3** The effect of NPs (0.1-3 mg/mL) on the viability of RBECs. **A-B** Cellular viability of RBECs after 24 h incubation with TR-BSA encapsulated non-tagged and PepH3-tagged NPs. **C-D** Cellular viability of RBECs after 24 h incubation with sdAb loaded non-tagged and PepH3-tagged NPs. Values presented are means ± SD; ***p < 0.001, ****p < 0.0001 compared to the control group; Statistical analysis: one-way ANOVA followed by Bonferroni post-test; n=6-12. con: control group treated with culture medium; N(TR-BSA): TR-BSA encapsulated non-tagged NPs; N-PepH3(TR-BSA): TR-BSA encapsulated PepH3-tagged NPs; N(sdAb): sdAb encapsulated non-tagged NPs; N-PepH3(sdAb): sdAb encapsulated PepH3-tagged NPs.

*
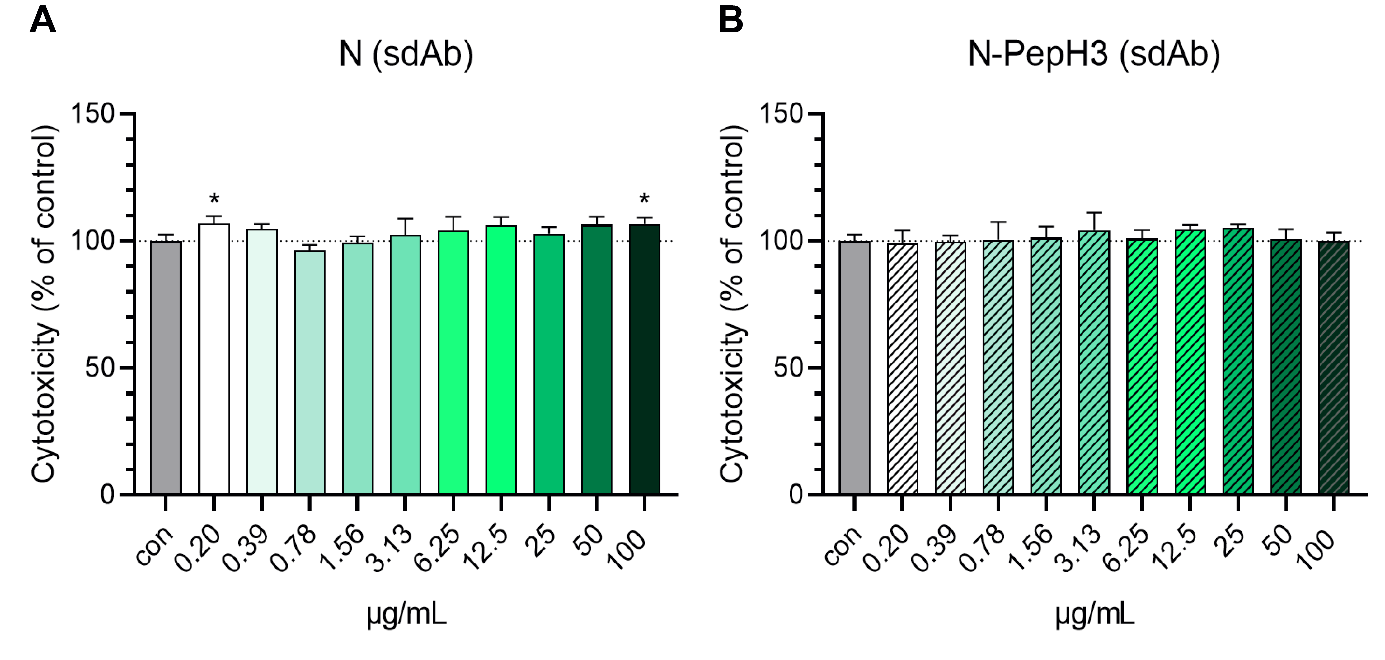
*

**Fig. S4** The effect of sdAb loaded non-tagged and PepH3-tagged NPs on the viability of HBEC-5i cell line. Values presented are means ± SD; *p < 0.05 compared to the control group; Statistical analysis: one-way ANOVA followed by Bonferroni post-test; n=3. con: control group treated with culture medium; N(sdAb): sdAb encapsulated non-tagged NPs; N-PepH3(sdAb): sdAb loaded PepH3-tagged NPs.


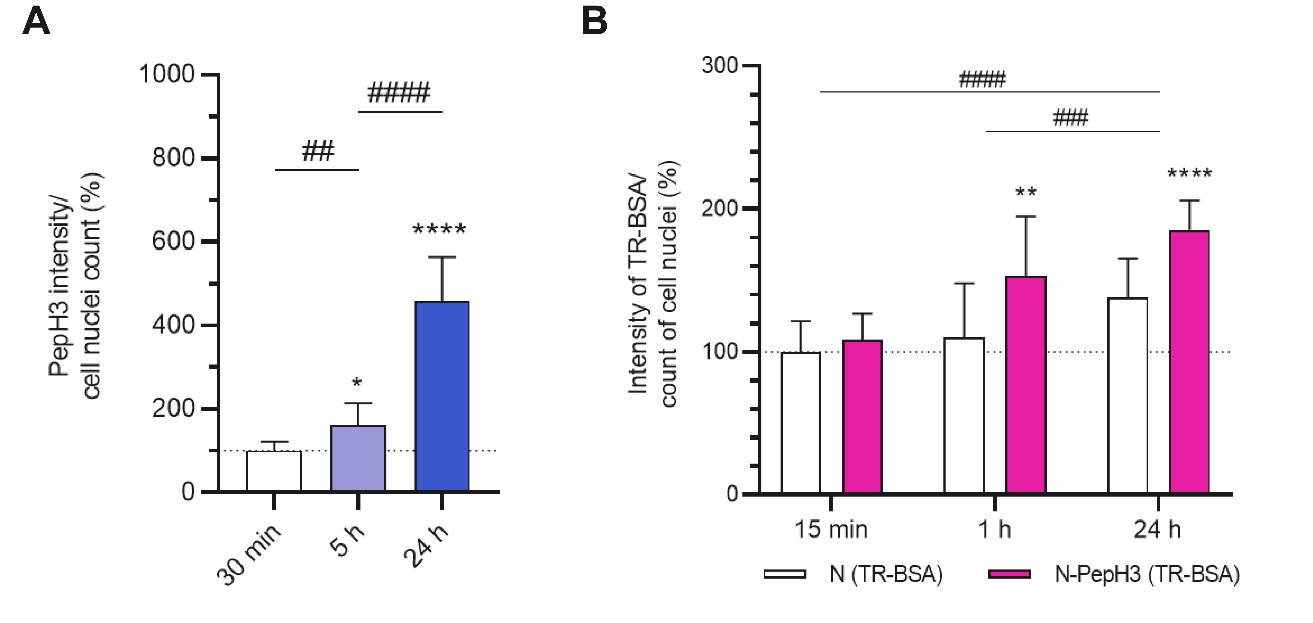


**Fig. S5** Time-dependent cellular uptake of the PepH3 peptide and NPs where the fluorescence intensity values were normalized to cell nuclei number. **A** Image analysis of cellular internalization of PepH3 peptide. The values are presented as percentage of the intensity values at the 30 min time point. Values presented are means ± SD. Statistical analysis: two-way ANOVA followed by Bonferroni post-test; ^*^p < 0.05, ^****^p < 0.0001 compared to 30 min group; ^##^p < 0.01, ^####^p < 0.0001 compared to 5 h group; n=10-25. **B** cellular internalization of Texas-Red loaded non-targeted and PepH3-tagged nanoparticles. The values are presented as percentage of the intensity values at the 15 min time point for untargeted NPs. Values presented are means ± SD; Statistical analyses: two-way ANOVA followed by Bonferroni post-test; ^**^p < 0.01, ^****^p < 0.0001 compared to N groups in each time point; ^##^p < 0.01, ^####^p < 0.0001 compared to 24 h time point; n=10-15. N: non-tagged nanoparticle; N-PepH3: PepH3-tagged nanoparticle; TR-BSA: Texas red-labelled bovine serum albumin.


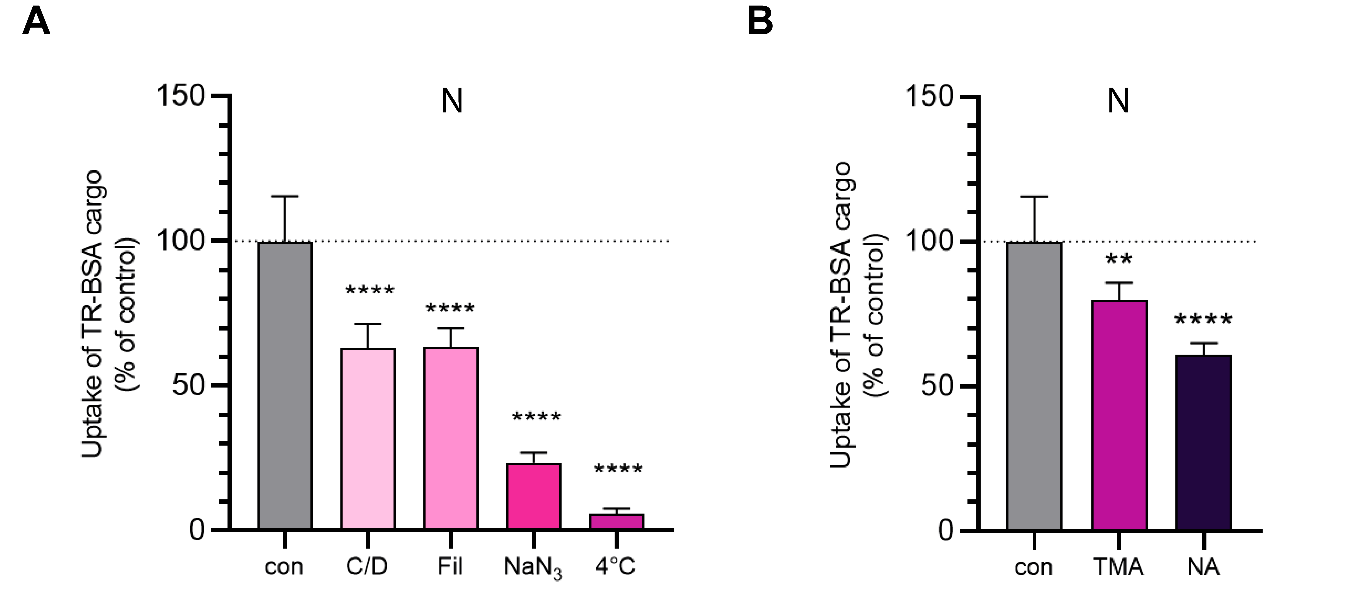


**Fig. S6** Cellular uptake mechanisms of the non-targeted TR-BSA loaded NPs. **A** The effect of endocytic inhibitor cytochalasin D (C/D; 0.125 μg/mL) and filipin (Fil; 5 μg/mL) or metabolic inhibitor sodium azide (NaN3; 1 mg/mL), and incubation at 4ºC on the cellular uptake of TR-BSA loaded non-targeted NPs (24 h). Values presented are means ± SD and are given as a percentage of the control groups. Statistical analysis: one-way ANOVA followed by Bonferroni post-test; ****p < 0.0001 compared to control group; n=6. **B** The effect of the modification of glycocalyx with neuraminidase (NA; 1 U/mL) and TMA-DPH (TMA; 30 mM) on the cellular uptake of TR-BSA loaded non-targeted (37ºC, 24 h) NPs. Values presented are means ± SD and are given as a percentage of the control group. Statistical analysis: one-way ANOVA followed by Bonferroni post-test; **p < 0.01, ****p < 0.0001 compared to control group; n=6. con: control group treated with non-targeted NP treatment alone at 37 ºC without any inhibitors.

*
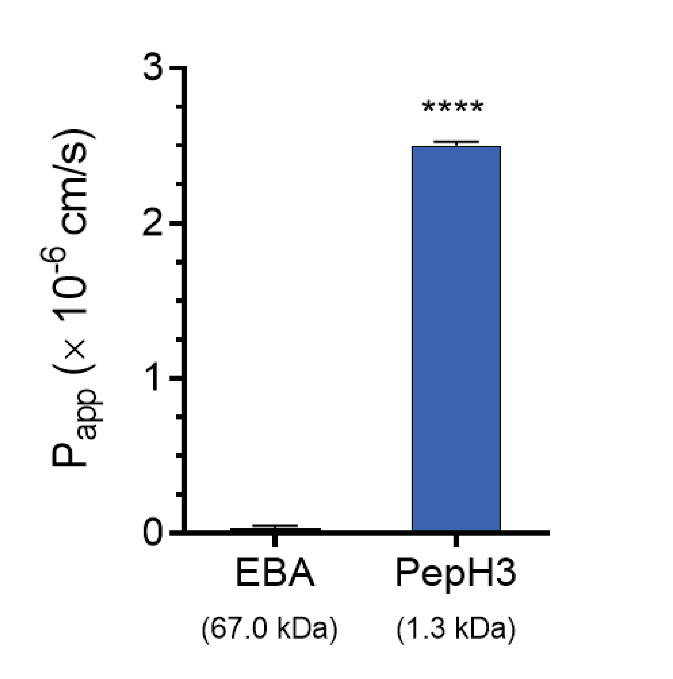
*

**Fig. S7** Permeability of Quasar 570 labelled PepH3 peptide (1.3 kDa, 10 nM, 30 min) and the BBB marker molecule, albumin (67 kDa) across the rat BBB model. Values presented are means ± SEM. Statistical analysis: unpaired t-test; ****p < 0.0001 compared to EBA; n=3. P_app_: apparent permeability coefficient; EBA: Evans-blue albumin.

*
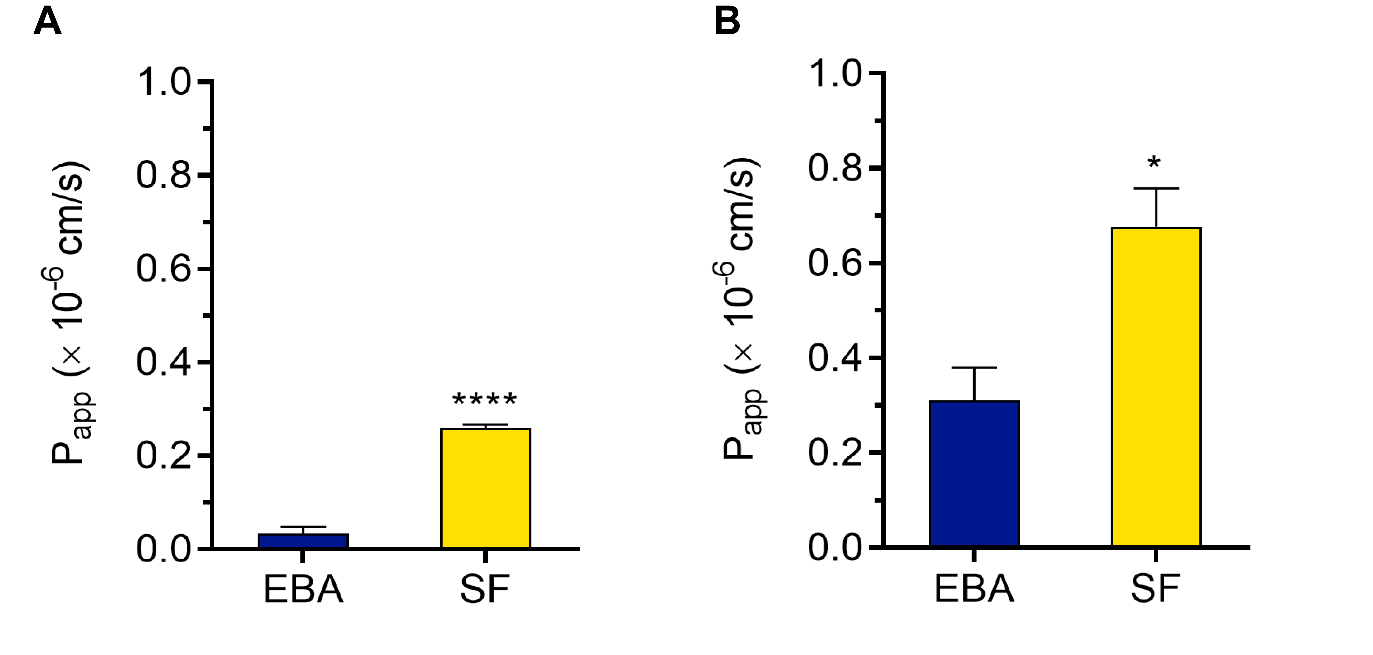
*

**Fig. S8** Permeability of transcellular BBB marker, EBA and the paracellular BBB marker, SF across the rat BBB model. **A** Permeability of EBA and SF after 30 min incubation in the experiment of PepH3. **B** Permeability of EBA and SF after 24 h incubation in the experiment of NPs with TR-BSA cargo. Values presented are means ± SD; *p < 0.05, ****p < 0.0001 compared to the EBA group; Statistical analysis: unpaired t-test; n=3-6. EBA: Evans-blue albumin; SF: sodium fluorescein.

***
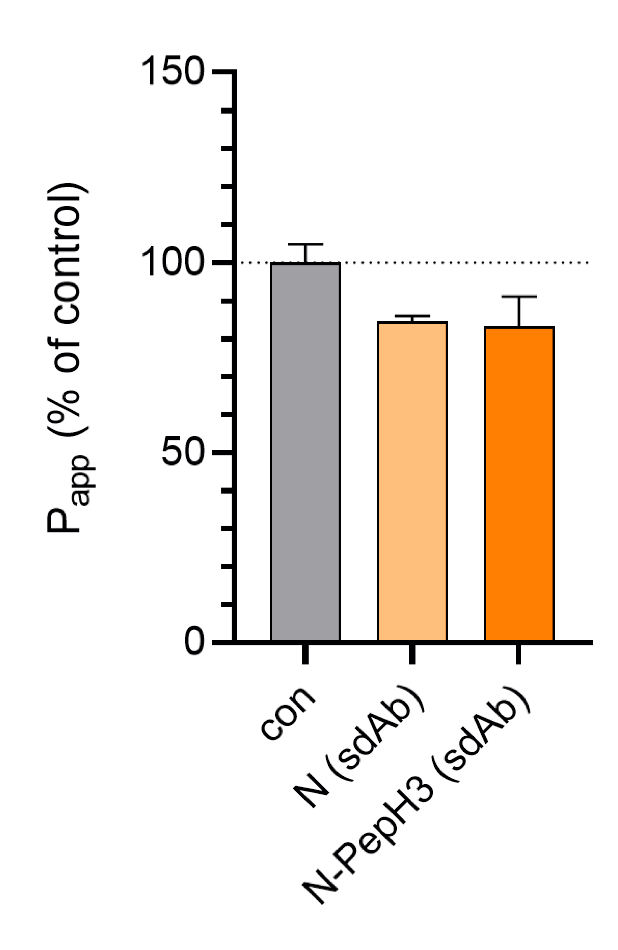
***

**Fig. S9** Permeability of the BBB marker FD4 (4 kDa) across the human BBB model after 2 h incubation of NPs with sdAb cargo. Values presented are means ± SD and are given as a percentage of the control group; Statistical analysis: one-way ANOVA followed by Bonferroni post-test; n=8. con: control group treated with culture medium; FD4: fluorescein isothiocyanate-4 kDa dextran.
